# Phytochemical Analysis Using LC–MS/MS and Anticancer Activity of *Asphodelus tenuifolius* Roots: Cell Cycle Arrest, Apoptosis, and In Silico Insights

**DOI:** 10.1002/cbdv.71724

**Published:** 2026-09-22

**Authors:** Racha Lydia Bouchouka, Farida Larit, Zahia Kabouche, Marlène Debiossat, Laurent Riou, Catherine Ghezzi, Francisco León, Ahmed Kabouche

**Affiliations:** ^1^ Laboratoire D'obtention de Substances Thérapeutiques (LOST) Université Constantine 1‐Frères Mentouri Constantine Algeria; ^2^ Université de Grenoble Alpes Grenoble France; ^3^ Department of Drug Discovery and Biomedical Sciences College of Pharmacy University of South Carolina Columbia South Carolina USA; ^4^ Université Constantine 1‐Frères Mentouri Constantine Algeria

**Keywords:** anticancer activity, apoptosis, *Asphodelus tenuifolius*, LC–HRMS/MS, molecular docking

## Abstract

*Asphodelus tenuifolius* Cav. (Asphodelaceae) is traditionally used in North African ethnomedicine for inflammatory and oxidative stress‐related disorders. In this study, the hydroethanolic root extract was investigated by LC–HRMS/MS, leading to the identification of 58 metabolites, mainly flavonoids, anthraquinones, and cinnamic acid derivatives. Major constituents included luteolin, emodin, luteolin‐7‐*O*‐β‐D‐glucoside, subaphyllin (*N*‐*trans*‐feruloylputrescine), and *N‐trans*‐feruloyltyramine. The extract showed moderate antiproliferative activity against MDA‐MB‐231 and PC‐3 cells, while flow cytometry revealed pronounced apoptosis induction (up to 85%) and significant G0/G1 cell‐cycle arrest. Molecular docking and target prediction suggested multitarget modulation of ERα, AR, AKT1, CDK2, and BCL‐2, particularly by aglycone metabolites. ADMET analysis indicated favorable drug‐likeness and safety profiles. These findings provide the first mechanistic evidence supporting the multitarget anticancer potential of *A. tenuifolius* roots and highlight their value as a source of bioactive anticancer scaffolds.

## Graphical abstract

LC–HRMS/MS profiling of the hydroethanolic root extract of *A. tenuifolius* identifies 58 metabolites, including luteolin, emodin, luteolin‐7‐*O*‐β‐D‐glucoside, subaphyllin, and N‐trans‐feruloyltyramine. Integrated biological and computational analyses reveal apoptosis induction, G0/G1 cell‐cycle arrest, and multi‐target interactions, highlighting the roots as a promising source of natural anticancer agents.

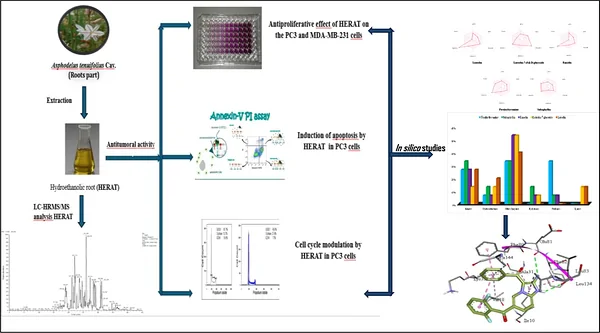

## 1 Introduction

Natural products remain a cornerstone of anticancer drug discovery, serving both as direct therapeutic agents and as privileged scaffolds for structural optimization [1, 2]. A substantial proportion of clinically approved anticancer drugs are derived from or inspired by natural compounds, underscoring their remarkable structural diversity and biological adaptability [2]. Among these, polyphenols, anthraquinones, and related phenolic derivatives have attracted considerable attention due to their ability to modulate key hallmarks of cancer, including cell proliferation, apoptosis, oxidative stress, and cell‐cycle progression [1, 3, 4]. Their multitarget mode of action is particularly advantageous in aggressive and therapy‐resistant malignancies [1, 3, 5].

Triple‐negative breast cancer (MDA‐MB‐231, RRID:CVCL_0062) and androgen‐independent prostate cancer (PC‐3, RRID:CVCL_0035) cell lines are representative examples of such aggressive cancer phenotypes [4]. MDA‐MB‐231 cell line is characterized by the absence of estrogen receptor (ER), progesterone receptor (PR), and human epidermal growth factor receptor 2 (HER2) expression, resulting in poor prognosis and limited options for targeted therapy. PC‐3 cell line exhibits resistance to androgen‐deprivation strategies through the dysregulation of survival kinases, cell‐cycle regulators, and apoptotic pathways [6, 7]. In this context, targeting interconnected signaling nodes such as protein kinase B alpha (AKT1), cyclin‐dependent kinase 2 (CDK2), B‐cell lymphoma 2 (BCL‐2), and hormone receptors represent a rational multitarget therapeutic strategy [8, 9, 10, 11]. Given the molecular complexity and adaptive resistance mechanisms that characterize both cancers, the identification of compounds capable of simultaneously modulating multiple oncogenic pathways has emerged as a promising approach to improve therapeutic efficacy and limit the development of resistance. Natural products are particularly attractive in this regard, as their structural diversity often confers intrinsic polypharmacological properties, enabling interactions with multiple molecular targets involved in tumor progression and survival.

The genus *Asphodelus* (Asphodelaceae), comprising approximately 20 species distributed throughout the Mediterranean region, Africa, and the Indian subcontinent, has long been recognized in ethnopharmacology for the treatment of acne, alopecia, burns, eczema, psoriasis, and inflammatory disorders [12, 13]. Toxicological investigations have demonstrated negligible toxicity, supporting its favorable safety profile [12]. Phytochemical studies have revealed a diverse array of secondary metabolites, including flavonoids, anthraquinones, and phenolic acids, which are associated with antioxidant, antimicrobial, and cytotoxic activities [12, 14, 15]. Among its species *A. tenuifolius* Cav., a desert species native to the Algerian Sahara, has traditionally been used to treat conditions associated with inflammation and oxidative stress [13, 16]. Recent studies have further highlighted its anti‐inflammatory, antithrombotic [13, 17], vasorelaxant, and antihypertensive properties [18]. Although the aerial parts of *A. tenuifolius* have been extensively investigated [4, 12, 13, 16, 18, 19], its roots remain comparatively underexplored, particularly regarding their metabolite composition and pharmacological potential, including anticancer mechanisms. Notably, Kabouche et al. [19] reported that extracts from the aerial parts of *A. tenuifolius* exhibited no significant anticancer activity across several cancer cell lines, thereby identifying the roots as an important and largely unexplored research gap.

Roots of many medicinal plants accumulate secondary metabolites that differ markedly from those present in aerial organs, often exhibiting higher concentrations of defense‐related compounds such as phenolic acids, anthraquinones, and alkaloids, whereas aerial tissues are generally enriched in flavonoids and light‐associated metabolites [20, 21]. These organ‐specific metabolic differences are closely linked to the distinct ecological functions of plant tissues. Continuously exposed to soil microorganisms, oxidative stress, and environmental challenges, roots frequently accumulate specialized defense‐related metabolites that are absent or present only at low concentrations in aerial organs. Consequently, root tissues often display unique phytochemical profiles and biological activities that cannot be predicted solely from studies conducted on leaves, stems, or flowers. Therefore, comprehensive organ‐specific phytochemical and pharmacological investigations are essential. This consideration is particularly relevant for species of the genus *Asphodelus*, whose roots are recognized as important reservoirs of anthraquinones and other phenolic metabolites frequently associated with cytotoxic, antiproliferative, and pro‐apoptotic activities. Consequently, the absence of significant anticancer activity in the aerial parts of *A. tenuifolius* does not preclude the possibility that biologically active constituents may be selectively accumulated in the roots. To date, however, the phytochemical composition and anticancer relevance of *A. tenuifolius* roots remain largely unexplored.

In this study, we investigated the hydroethanolic root extract of *A. tenuifolius* (HERAT) as a promising source of anticancer metabolites. Although this species has demonstrated considerable pharmacological potential, its root metabolites have not been comprehensively characterized or evaluated using integrated metabolomic and computational approaches, limiting the identification of bioactive compounds and their molecular targets. We hypothesized that HERAT contains metabolites capable of modulating key oncogenic pathways involved in tumor progression and survival. To test this hypothesis, LC–HRMS/MS‐based metabolic profiling was combined with computational pharmacology to characterize the phytochemical composition of HERAT, predict pharmacokinetic and target profiles, and prioritize bioactive constituents. The extract was further evaluated for its antiproliferative effects against prostate (PC‐3) and triple‐negative breast (MDA‐MB‐231) cancer cells, as well as its ability to induce apoptosis and alter cell‐cycle progression. Molecular docking and complementary in silico target prediction analyses were performed to identify potential molecular targets and elucidate the mechanisms underlying its anticancer activity.

This integrated experimental–computational strategy constitutes the first comprehensive investigation of the roots of *A. tenuifolius*, a plant organ that remains largely unexplored compared with the aerial parts. Beyond the experimental evaluation, the computational workflow enabled the annotation of bioactive root metabolites, the assessment of their drug‐likeness and pharmacokinetic (ADMET) properties using SwissADME [22], the prediction of their putative protein targets through SwissTargetPrediction [22], and the evaluation of their binding affinities by molecular docking using AutoDock Vina [23] against experimentally resolved crystallographic protein structures. Docking analyses were performed on five key cancer‐related targets, namely estrogen receptor alpha (ERα), androgen receptor (AR), protein kinase B (AKT1), cyclin‐dependent kinase 2 (CDK2), and B‐cell lymphoma 2 (BCL‐2), all of which play critical roles in breast and prostate cancer progression.

Accordingly, this study was designed to bridge the existing knowledge gap regarding the phytochemical composition and anticancer potential of *A. tenuifolius* roots. By combining untargeted LC–HRMS/MS metabolite profiling with computational pharmacology approaches, we sought to identify bioactive metabolites with favorable drug‐like properties and potential multitarget activities against biologically relevant proteins implicated in cancer development. Collectively, the findings provide the first comprehensive characterization of the metabolite profile of HERAT and offer mechanistic insights into its anticancer potential. These results establish a robust scientific basis for future biological validation and support the development of *A. tenuifolius*‐derived metabolites as promising lead candidates for anticancer drug discovery.

## 2 Results and Discussion

The anticancer potential of HERAT was investigated through an integrated chemical–biological approach combining LC–HRMS/MS metabolite profiling, in vitro mechanistic assays, and structure‐based computational analyses. This strategy aimed to correlate the identified secondary metabolites with their potential contribution to the observed cellular effects.

LC–HRMS/MS analysis enabled the annotation of 58 metabolites, predominantly flavonoids, anthraquinones, and phenolic derivatives. Major constituents included luteolin, luteolin‐7‐*O*‐β‐D‐glucoside, emodin, subaphyllin, and *N‐trans*‐feruloyltyramine. These classes of compounds are widely recognized for their ability to modulate proliferation and apoptosis‐related pathways [2, 24], suggesting that the biological activity of the extract may result from a combined or synergistic effect of multiple bioactive scaffolds rather than from a single dominant compound.

Given the complexity of the phytochemical profile and the multitarget nature of the observed biological responses, an in silico pharmacological analysis was undertaken to identify plausible molecular mediators. Target prediction, ADMET assessment, and molecular docking analysis were employed to explore interactions between major metabolites and key proteins involved in survival signaling, hormone receptor activity, kinase regulation, and mitochondrial apoptosis.

The following sections discuss the chemical characterization of the extract and systematically correlate the experimental findings with computational insights to propose a mechanistic framework underlying the multitarget modulation profile of *A. tenuifolius* root metabolites.

### 2.1 LC‐HRMS/MS Analyses of HERAT

LC‐HRMS/MS analysis of the HERAT revealed a chemically diverse metabolite profile, with the identification of major constituents belonging to several classes of bioactive secondary metabolites (Table 1 and Figure S1). The most abundant annotated compounds included luteolin (18.51%), subaphyllin (14.04%), emodin (10.45%), luteolin‐7‐*O*‐β‐D‐glucoside (11.86%), and feruloyltyramine (8.20%), based on relative peak area normalization.

**TABLE 1:** LC–HRMS/MS analysis of compounds identified in the hydroethanolic root extract of *A. tenuifolius* Cav. (HERAT).

**No**.	**Compounds**	**Rt (min)** ^a^	***m/z* (molecular ion)**	**Major fragments *m/z* (intensity)**	**Chemical Class**	**Relative abundance (%)**	**SMILE**	**CAS**
**1**	2',3'‐Epoxyindicolactone	4.14	383.1147		Furocoumarin	0.04	CC1 = CC(OC1 = O)CC2(C(O2)COC3 = C4C(= CC5 = C3OC = C5)C = CC(= O)O4)C	312619‐44‐6
**2**	Melibiose	4.35	365.1042	343.1299 (100), 325.1184 (75), 307.1070 (50), 289.0955 (45), 171.0596 (30	Furocoumarin	0.91	C(C1C(C(C(C(O1)OCC2C(C(C(C(O2)O)O)O)O)O)O)O)O	5340‐95‐4
**3**	Adenosine	10.24	268.1030	136.0618 (100), 268.1033 (45), 119.0504 (20), 148.0616 (15)	Purine nucleoside	1.56	C1 = NC(= C2C(= N1)N(C = N2)C3C(C(C(O3)CO)O)O)N	58‐61‐7
**4**	4‐*p*‐Coumaroylquinic acid	17.31	339.1067	163.0392 (110), 119.0504 (37)	Cinnamic acid	0.01	C1C(C(CC1(C(= O)O)O)O)OC(= O)C = C/C2 = CC = C(C = C2)O)O	1108200‐72‐1
**5**	Chlorogenic acid	17.60	355.1015	163.0300 (100)	Cinnamic acid	3.17	C1C(C(C(CC1(C(= O)O)O)OC(= O)C = CC2 = CC(= C(C = C2)O)O)O)O	202650‐88‐2
**6**	Aloesin	17.85	395.1325	336.0845 (100)	Chromone	0.19	CC1 = CC(= C(C2 = C1C(= O)C = C(O2)CC(= O)C)C3C(C(C(C(O3)CO)O)O)O)O	30861‐27‐9
**7**	2‐[(6‐Methoxyquinolin‐8‐yl) amino]propanoic acid	18.23	247.1071	247.1077(100), 216.0814 (35), 188.0709 (22), 159.0552 (18), 121.0448 (12)	Anthranilic acid alkaloid	0.06	COC1 = CC(= C2C(= C1)C = CC = N2)N	90‐52‐8
**8**	8‐β‐D‐Glucosyl‐5,7‐dihydroxy‐2‐(2‐methylpropyl) chromen‐4‐one	18.60	397.1470	235.0912 (80), 217.0805 (65), 191.0600 (50)	Chromone	0.41	COC1 = C(C = C(C = C1)C2 = CC(= O)C3 = C(C = C(C = C3O2)O)O)C4 = C(C = C(C5 = C4OC(= CC5 = O)C6 = CC = C(C = C6)O)O)OC	41583‐84‐0
**9**	1,2,3,4‐Tetrahydro‐β‐carboline‐3‐carboxylic acid	18.99	231.1136	205.0972(100), 188.0709 (42), 160.0604 (25), 144.0448 (20), 130.0648 (15)	Carboline alkaloid	0.25	CC1C2 = C(CC(N1)C(= O)O)C3 = CC = CC = C3N2	5470‐37‐1
**10**	Vicenin 2	19.13	595.1651	503.1234 (24), 437.1052 (100) 382.0768(100), 353.0645 (100)	Flavone	0.11	C1 = CC(= CC = C1C2 = CC(= O)C3 = C(C(= C(C(= C3O2)C4C(C(C(C(O4)CO)O)O)O)O)C5C(C(C(C(O5)CO)O)O)O)O)O	23666‐13‐9
**11**	3‐*p*‐Coumaroylquinic acid	19.20	339.1068	163.0392(110), 119.0504 (37)	Cinnamic acid	0.49	C1C(C(C(CC1(C(= O)O)O)OC(= O)/C = C/C2 = CC = C(C = C2)O)O)O	87099‐71‐6
**12**	*N*‐Methyl‐2,4‐dihydroxy‐3‐phenylquinoline	19.37	252.0863	268.0968(100), 253.0732 (35), 225.0627 (20), 210.0391 (18), 105.0335 (15)	Quiline alkaloid	0.02	CN1C2 = CC = CC = C2C(= C(C1 = O)C3 = CC = CC = C3)O	519‐66‐4
**13**	5‐Feruloylquinic acid	19.49	367.1020	163.0389(100), 324.3259 (95), 145.0284 (79)	Cinnamic acid amide	0.98	COC1 = C(C = CC(= C1)C = CC(= O)OC2CC(CC(C2O)O)(C(= O)O)O)O	1899‐29‐2
**14**	Methyl chlorogenate	19.59	369.1172	177.0536(100), 117.0327 (94), 145.0277 (70), 149.0594 (55)	Cinnamic acid	2.21	COC(= O)C1(CC(C(C(C1)OC(= O)C = CC2 = CC(= C(C = C2)O)O)O)O)O	123483‐19‐2
**15**	8‐*O*‐Acetylharpagide	19.78	429.1359	369.116 (100), 351.106 (95.7), 203.054 (49.7), 370.120 (14.4)	Iridoid glycoside	0.05	CC(= O)OC1(CC(C2(C1C(OC = C2)OC3C(C(C(C(O3)CO)O)O)O)O)O)C	6926‐14‐3
**16**	Quercetin 3‐*O*‐gallate	20.17	617.1126	301 (100), 179 (45), 151 (30), 107 (15)	Flavonol	0.009	C1 = CC(= C(C = C1C2 = C(C(= O)C3 = C(C = C(C = C3O2)O)O)O)OC4C(C(C(C(O4)CO)O)O)OC(= O)C5 = CC(= C(C(= C5)O)O)O)O	53209‐27‐1
**17**	Myricetin 3‐*O*‐β‐D‐galactoside	20.35	481.0964	319.0442 (100)	Flavonol	0.002	C1 = C(C = C(C(= C1O)O)O)C2 = C(C(= O)C3 = C(C = C(C = C3O2)O)O)OC4C(C(C(C(O4)CO)O)O)O	15648‐86‐9
**18**	Peltatoside (quercetin‐3‐*O*‐arabinoglucoside)	20.59	597.1439	303.0562(100), 85.0232 (30)	Flavonol	0.10%	C1C(C(C(C(O1)OCC2C(C(C(C(O2)OC3 = C(OC4 = CC(= CC(= C4C3 = O)O)O)C5 = CC(= C(C = C5)O)O)O)O)O)O)O)O	23284‐18‐6
**19**	Sayaendoside	20.62	439.1569	417.1443 (100), 399.1338 (80), 381.1233 (60), 163.0385 (40)	Phenylethaoid	0.23	C1C(C(C(O1)OCC2C(C(C(C(O2OCCC3 = CC = CC = C3)CO)O)O)O)(CO)O	371113‐07‐4
**20**	Panasenoside	21.00	611.1595	447 (100), 285 (68), 255 (35), 227 (18)	Flavonol	0.51	C1 = CC(= CC = C1/C = C/C(= C∖2/C(= C(C(= O)C(C2 = O)(C3C(C(C(C(O3)CO)O)O)O)O)(C4C(C(C(C(O4)CO)O)O)O)/O)O	78281‐02‐4
**21**	Hyperoside	21.01	465.1015	301 (100), 179 (45), 151 (20)	Flavonone	0.16	C1 = CC(= C(C = C1C2 = C(C(= O)C3 = C(C = C(C = C3O2)O)O)OC4C(C(C(C(O4)CO)O)O)O)O)O	482‐36‐0
**22**	2‐Phenylethyl 6‐*O*‐β‐D‐xylopyranosyl‐β‐D‐glucoside.	21.14	439.1565		Phenylethaoid	0.034	C1C(C(C(C(O1)OCC2C(C(C(C(O2)OCCC3 = CC = CC = C3)O)O)O)O)O)O	129932‐48‐5
**23**	Rutin	21.15	611.1592	301 (100), 271 (42), 255 (28), 179 (15)	Flavonol	0.14	CC1(C(C(C(C(O1)OCC2C(C(C(C(O2)OC3 = C(OC4 = CC(= CC(= C4C3 = O)O)O)C5 = CC(= C(C = C5)O)O)O)O)O)O)O)O	153‐18‐4
**24**	(2E,4E) ‐5‐{6‐[(β‐D‐glucosyloxy) methyl] ‐1‐hydroxy‐2,6‐dimethyl‐4‐oxo‐2‐cyclohexen‐1‐yl}‐3‐methyl‐2,4‐pentadienoic acid	21.44	443.1894		Apocarotenoid	0,03	CC1 = C(C(CCC1)(C)C)/C = C/C(= C/C(= O)O)/C	14398‐42‐6
**25**	Isovitexin	21.47	433.1119	415.1024 (98), 313.0705 (31)	Flavone	0.001	C1 = CC(= CC = C1C2 = CC(= O)C3 = C(O2)C = C(C(= C3O)C4C(C(C(C(O4)CO)O)O)O)O)O	38953‐85‐4
**26**	Naringenin‐7‐*O*‐β‐D‐glucoside	21.63	435.1274	273.0759 (100), 153.0193 (65) 147.0451 (28)	Flavonone	0.04	C1C(OC2 = CC(= CC(= C2C1 = O)O)OC3C(C(C(C(O3)CO)O)O)O)C4 = CC = C(C = C4)O	529‐55‐5
**27**	Apigenin 6,8‐digalactoside	21.72	593.1486	431.0975 (80), 269.0458 (60), 153.0185 (40)	Flavone	0.001	C1 = CC(= CC = C1C2 = CC(= O)C3 = C(C(= C(C(= C3O2)C4C(C(C(C(O4)CO)O)O)O)O)C5C(C(C(C(O5)CO)O)O)O)O)O	82043‐11‐6
**28**	Orientin	21.77	447.0913	445.0777 (85), 895.1959 (41)	Flavone	0.51	C1 = CC(= CC = C1C2 = C(C(= O)C3 = C(C = C(C = C3O2)O)O)O)OC4C(C(C(C(O4)CO)O)O)O	480‐10‐4
**29**	Kaempferol‐3‐*O*‐rutinoside	21.78	595.1647	433.1135 (85), 287.0550 (70), 153.0185 (50)	Flavonol	0.16	CC1C(C(C(C(O1)OCC2C(C(C(C(O2)OC3 = C(OC4 = CC(= CC(= C4C3 = O)O)O)C5 = CC = C(C = C5)O)O)O)O)O)O)O	17650‐84‐9
**30**	Luteolin‐7,3'‐di‐*O*‐glucoside	22.12	609.1444	449.1095 (100), 451.1146 (38)	Flavone	0.43	C1 = CC(= C(C = C1C2 = CC(= O)C3 = C(C = C(C = C3O2)OC4C(C(C(C(O4)CO)O)O)O)O)OC5C(C(C(C(O5)CO)O)O)O)O	52187‐80‐1
**31**	Luteolin‐7‐*O*‐β‐D‐glucoside	22.16	449.1066	287.0556 (100), 431.0978 (47)	Flavone	1.95	C1 = CC(= C(C = C1C2 = CC(= O)C3 = C(C = C(C = C3O2)OC4C(C(C(C(O4)CO)O)O)O)O)O)O	1268798
**32**	3’‐Methoxyqueretin‐3‐*O*‐rutinoside	22.33	625.1745	315 (100), 300 (62), 271 (35), 151 (18)	Flavonol	0.003	CC1C(C(C(C(O1)OCC2C(C(C(C(O2)OC3 = C(OC4 = CC(= CC(= C4C3 = O)O)O)C5 = CC(= C(C = C5)O)OS(= O)(= O)O)O)O)O)O)O)O.[	1486‐70‐0
**33**	Chrysoeriol 7‐*O* neohesperidosyl	22.38	609.1804	447.1285 (80), 431.0975 (60), 285.0754 (50), 151.0481 (30)	Flavone	2.28	CC1C(C(C(C(O1)OC2C(C(C(O[C]2OC3 = CC(= C4C(= C3)OC(= CC4 = O)C5 = CC(= C(C = C5)O)OC)O)CO)O)O)O)O)O	111133‐90‐5
**34**	Roseoside	22.43	387.2001	419.1254(100), 285.0843 (42), 267.0738 (35), 179.0456 (25), 151.0364 (18)	Megastigmane	0,03	CC1 = CC(= O)CC(C1(C = CC(C)OC2C(C(C(C(O2)CO)O)O)O)O)(C)C	54835‐70‐0
**35**	**Subaphyllin**	**23.01**	**314.1379**	**235.1702 (100), 217.1589 (60), 189.1484 (45), 161.1379 (30), 133.1274 (25)**	**phenolic amide**	**14,04**	**COC1 = C(C = CC(= C1)C = CC(= O)NCCCCN)O**	**501‐13‐3**
**36**	Tiliroside	23.04	593.1490	309.0992 (84), 291.0894 (54) 287.0563(100), 147.0459 (100) 119.0505 (98)	Flavanol	0.62	C1 = CC(= CC = C1C = CC(= O)OCC2C(C(C(C(O2)OC3 = C(OC4 = CC(= CC(= C4C3 = O)O)O)C5 = CC = C(C = C5)O)O)O)O)O	20316‐62‐5
**37**	**Luteolin‐7‐*O*‐β‐D‐glucoside** **isomer**	**23.18**	**447.0915**	**285.0393 (100), 153.0177 (45)**	**Flavone**	**11.86**	**C1 = C(C = C(C(= C1O)O)O)C2 = C(C(= O)C3 = C(C = C(C = C3O2)O)O)OC4C(C(C(C(O4)COC(= O)C5 = CC(= C(C(= C5)O)O)O)O)O)O**	**15648‐86‐9**
**38**	Byzantionoside B	23.72	373.2196	419.1254 (100), 285.0843 (55), 267.0738 (40), 179.0456 (30), 151.0364 (20)	Apocarotenoid	0.60	CC1 = CC(= O)CC(C1CC(C)O C2C(C(C(C(O2)CO)O)O)O)(C)C	135820‐80‐3
**39**	(2R,3S,4S,5R,6S) ‐2‐(hydroxymethyl) ‐6‐[4‐(hydroxymethyl) ‐1‐propan‐2‐ylcyclohex‐3‐en‐1‐yl] oxyoxane‐3,4,5‐triol	23.85	355.1718	185 (100), 167 (72), 149 (45), 131 (21)	Menthane monoterpenoid	0.08	CC(C)C1(CCC(= CC1)CO)OC2C(C(C(C(O2)CO)O)O)O	16203‐27‐3
**40**	Diosmin	24.36	607.1649	430.0987 (100), 281.0437 (90)	Flavone	1.08	CC1C(C(C(C(O1)OCC2C(C(C(C(O2)OC3 = CC(= C4C(= C3)OC(= CC4 = O)C5 = CC(= C(C = C5)OC)O)O)O)O)O)O)O)O	520‐27‐4
**41**	Scoparone‐5‐*O*‐β‐D‐glucoside.	24.45	461.1070	321.0906 (100), 179.0456 (45), 163.0601 (35), 131.0406 (20), 105.0335 (18)	Coumarin	1.53	COC1 = CC2 = C(C(= CC(= O)O2)C3 = CC(= C(C = C3)O)O)C(= C1)OC4C(C(C(C(O4)CO)O)O)O	
**42**	**Luteolin**	24.68	287.0543	153.0182(65.44), 241.0497 (18), 245.0497 (17)	Flavones	**18.51**	**C1 = CC(= C(C = C1C2 = CC(= O)C3 = C(C = C(C = C3O2)O)O)O)O**	**491‐70‐3**
**43**	Nematophin	24.66	273.1648	347.2670(100), 329.2555 (55), 271.2083 (40), 247.1769 (30), 220.1457 (25)	Simple indole alkaloid	0.09	CCC(C)C(= O)C(= O)NCCC1 = CNC2 = CC = CC = C21	183314‐24‐1
**44**	**Feruloyltyramine**	**25.02**	**312.1231**	**295.0958 (80), 277.0853 (60), 219.0600 (40), 191.0495 (30)**	**Cinnamic acid** **amide**	**8.20**	**COC1 = C(C = CC(= C1)C = CC(= O)NCCC2 = CC = C(C = C2)O)O**	
**45**	Coumarin	25.03	667.1860	507.1397 (80), 345.0872 (60), 330.0634 (50), 315.0401 (40), 300.0273 (30)	Flavone	0.03	CC(= O)OCC1C(C(C(C(O1)OC2C(C(C(OC2OC3 = C(C4 = C(C(= C3)O)C(= O)C = C(O4)C5 = CC = C(C = C5)OC)O)CO)O)O)O)O)O	80680‐48‐4
**46**	9‐(2,3‐dihydroxypropoxy) ‐9‐oxononanoic acid	25.22	261.1336	171 (100), 153 (68), 127 (42), 99 (20)	Hydroxy fatty acid	0.46	C(CCCC(= O)O)CCCC(= O)OCC(CO)O	109421‐77‐4
**47**	Kaempferol‐3‐*O*‐ β‐D‐ glucoside	25.26	447.0916	287.0549(44), 431.0975 (20), 383.0761 (16)	Flavonol	1.22	C1 = CC(= CC = C1C2 = C(C(= O)C3 = C(C = C(C = C3O2)O)O)OC4C(C(C(C(O4)CO)O)O)O)O	480‐10‐4
**48**	Apigenin‐7‐*O*‐ β‐D‐ glucoside	25.36	431.0966	117.0351 (100), 269.0458 (58), 311.0519 (60)	Flavone	0.02	C1 = CC(= CC = C1C2 = CC(= O)C3 = C(C = C(C = C3O2)OC4C(C(C(C(O4)CO)O)O)O)O)O	578‐74‐5
**49**	Geranyl 6‐*O*‐α‐L‐arabinofuranosyl‐*O*‐β‐D‐ glucoside	25.96	471.2197	419.1912(100), 285.1256 (50), 267.1151 (40), 179.0865 (30), 151.0764 (20)	Acyclic monoterpenoid	0.01	CC(= CCC/C(= C/COC1C(C(C(C(O1)COC2C(C(C(O2)CO)O)O)O)O)O)/C)C	84534‐32‐7
**50**	(R)‐linalyl‐β‐vicianoside	26.20	471.2194	301.1684 (100), 283.1568 (55), 255.1463 (40), 227.1362 (30), 119.0719 (20)	Acyclic monoterpenoid	0.01	CC(= CCC(C)(C = C)OC1C(C(C(C(O1)COC2C(C(C(CO2)O)O)O)O)O)O)C	
**51**	6,8‐dihydroxy‐3‐(10‐hydroxyundecyl) ‐3,4‐dihydroisochromen‐1‐one	26.98	351.2133		Isocoumarin	2.90	CC(CCCCCCCCCC1CC2 = C(C(= CC(= C2)O)O)C(= O)O1)O	154850‐35‐8
**52**	Lauryl diethanolamide | *N*, *N*‐bis(2‐hydroxyethyl) dodecanamide	27.88	288.2525		*N*‐acyl ethanolamine	1,06	CCCCCCCCCCCC(= O)N(CCO)CCO	120‐40‐1
**53**	Phytosphingosine	28.69	318.2994		Sphingoid base	3.28	CCCCCCCCCCCCCCC(C(C(CO)N)O)O	554‐62‐1
**54**	Afzelin	28.77	431.0968	319.0830 (100), 301.0715 (65), 273.0610 (50), 255.0505 (40), 151.0348 (20)	Flavanol	0.26	CC1C(C(C(C(O1)OC2 = C(OC3 = CC(= CC(= C3C2 = O)O)O)C4 = CC = C(C = C4)O)O)O)O	482‐39‐3
**55**	**Emodin**	**28.85**	**269.0447**	**271.0904 (100), 253.0789 (80), 225.0684 (60), 197.0579 (50), 179.0474 (40)**	**Anthraquinone** **and anthrone**	**10.45**	**CC1 = CC2 = C(C(= C1)O)C(= O)C3 = C(C2 = O)C = C(C = C3O)O**	**518‐82‐1**
**56**	2',4',6'‐Trihydroxydihydrochalcone	30.78	257.0811		Chalcone	1.32	C1 = CC = C(C = C1)C(= O)C = CC2C(C = C(C = C2O)O)O	1088‐08‐0
**57**	Tetrahydroxy‐flavone	31.12	285.0394	303.0626 (100), 285.0511 (60), 257.0406 (45), 229.0301 (35), 151.0348 (20)	Flavanol	0.42	C1 = CC(= CC = C1C2 = C(C(= O)C3 = C(O2)C(= CC(= C3)O)O)O)O	3440‐24‐2
**58**	Pyrenophorol	31.41	311.1673	245.0797 (100), 227.0682 (70), 199.0577 (50), 171.0472 (40), 143.0367 (30)	Macrolide lactone	4.79	CC1CCC(C = CC(= O)OC(CCC(C = CC(= O)O1)O)C)O	22248‐41‐5

*Note*: Compounds shown in bold represent the major constituents identified in HERAT based on relative abundance.

^a^ Retention time.

Overall, the metabolite composition of HERAT was dominated by flavonoids, anthraquinones, and nitrogen‐containing phenolic derivatives, classes well known for their pharmacological relevance. Flavonoids, such as luteolin and its glycosylated derivatives, are widely reported for their antiproliferative, pro‐apoptotic, and cell‐cycle‐modulating activities. In contrast, anthraquinones, like emodin, have been associated with apoptosis induction and mitochondrial dysfunction in cancer cells. The presence of feruloyltyramine further suggests potential antioxidant and cytoprotective properties.

The chemical diversity and relative abundance of these metabolites provide a plausible molecular basis for the biological effects observed in subsequent in vitro assays. Rather than a single dominant compound, the pharmacological activity of HERAT is likely attributable to the synergistic action of multiple constituents. This complex phytochemical profile supports further mechanistic investigation of HERAT and highlights its potential relevance in the context of natural product–based anticancer research.

### 2.2 Anticancer Effects (Inhibition of Cell Proliferation, Induction of Apoptosis, and Cell Cycle Arrest)

#### 2.2.1 Blocking of Cell Proliferation

The antiproliferative activity of HERAT was evaluated against prostate cancer (PC‐3) and triple‐negative breast cancer (MDA‐MB‐231) cell lines. Cells were exposed to increasing concentrations of HERAT for 72 h, and cell viability was subsequently determined using the MTT assay.

**FIGURE 1:**
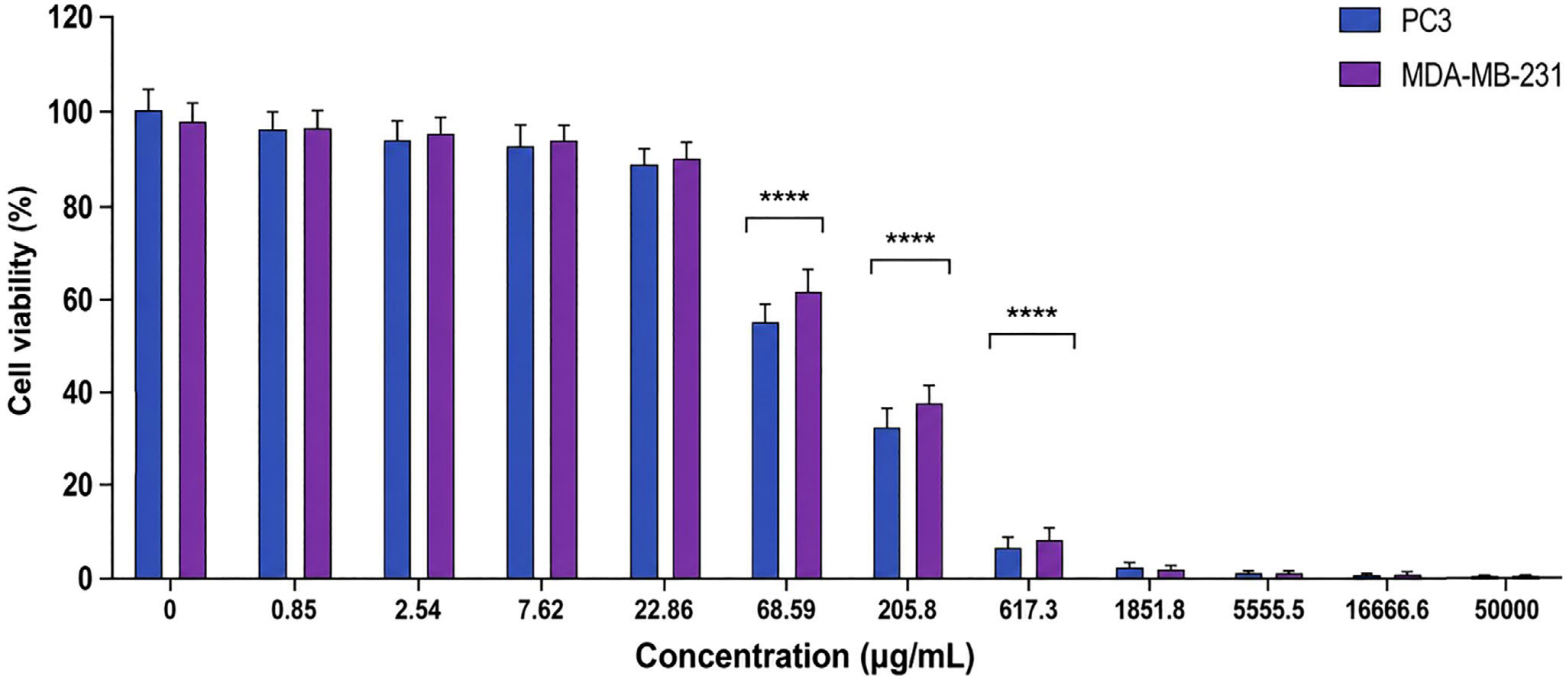
Dose‐dependent cytotoxic effects of HERAT on PC‐3 (RRID:CVCL_0035) and MDA‐MB‐231 (RRID:CVCL_0062) cells.

The hydroethanolic extract of HERAT exhibited moderate cytotoxic activity against the tested human cancer cell lines. The extract showed comparable inhibitory effects toward both PC‐3 prostate adenocarcinoma cells (RRID: CVCL_0035) and MDA‐MB‐231 breast adenocarcinoma cells (RRID: CVCL_0062), with IC_50_ values of 202.61 ± 5.40 and 200.08 ± 2.08 µg/mL (Table 2), respectively. The slightly lower IC_50_ value observed against MDA‐MB‐231 cells suggests a marginally higher sensitivity of breast cancer cells to the extract. A concentration‐dependent decrease in cell viability was observed, with statistically significant inhibition at higher extract concentrations (Figure 1 and Table S1).

**TABLE 2:** IC_50_ values (ug/mL) of HERAT against PC3 and MDA‐MB‐231 cancer cell lines.

IC_50_ values (µg /mL)^a^		
Cell type	HERAT^b^	Cisplatin^c^
PC3	202.61 ± 5.40	107 ± 4.02
MDA‐MB‐231	200.08 ± 2.08	112 ± 2.75

^a^ The data are presented as the mean ± standard deviation of IC50 (µg/mL) from three independent experiments.

^b^ Hydroethanolic root extract of *Asphodelus tenuifolius*.

^c^ Standard.

Overall, these findings indicate that HERAT possesses moderate antiproliferative potential, which may be attributed to the presence of bioactive phytochemicals such as phenolic compounds and flavonoids previously reported in *Asphodelus* species. Consequently, apoptosis induction and cell‐cycle modulation were examined to clarify whether the observed growth inhibition was associated with programmed cell death and regulatory effects on cell‐cycle progression.

Cell viability was assessed using the MTT assay and expressed as a percentage of the untreated control. PC‐3 and MDA‐MB‐231 cells were exposed to increasing concentrations of HERAT for 72 h. Data are presented as the mean ± standard deviation (SD) of three independent experiments (*n* = 3). Statistical analysis was performed using two‐way analysis of variance (ANOVA) followed by Bonferroni's post hoc test. Statistical significance was defined as ****p < 0.0001 compared with the control group.

#### 2.2.2 Apoptosis Induction

The pro‐apoptotic effect of HERAT was evaluated by flow cytometry following 24 h of treatment at different concentrations (250, 500, and 1000 µg/mL). Cells were stained with Annexin V–FITC and PI to distinguish viable cells, early apoptotic cells (Annexin V^+^/PI^−^), and late apoptotic or necrotic cells (Annexin V^+^/PI^+^). Total apoptosis was calculated as the sum of early and late apoptotic populations.

**FIGURE 2:**
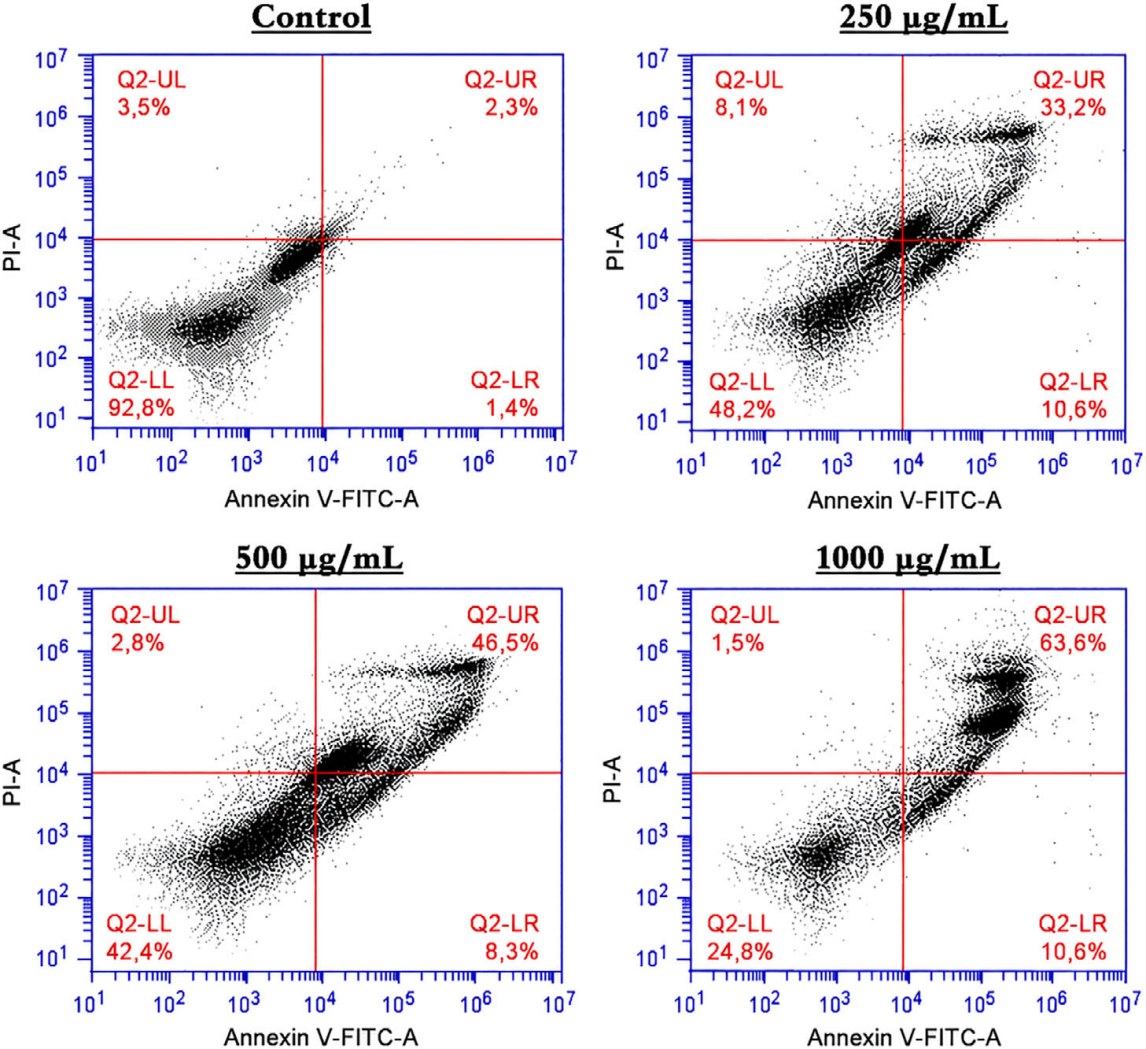
Apoptosis induction by HERAT in PC‐3 (RRID: CVCL_0035) cells analyzed by flow cytometry.

**FIGURE 3:**
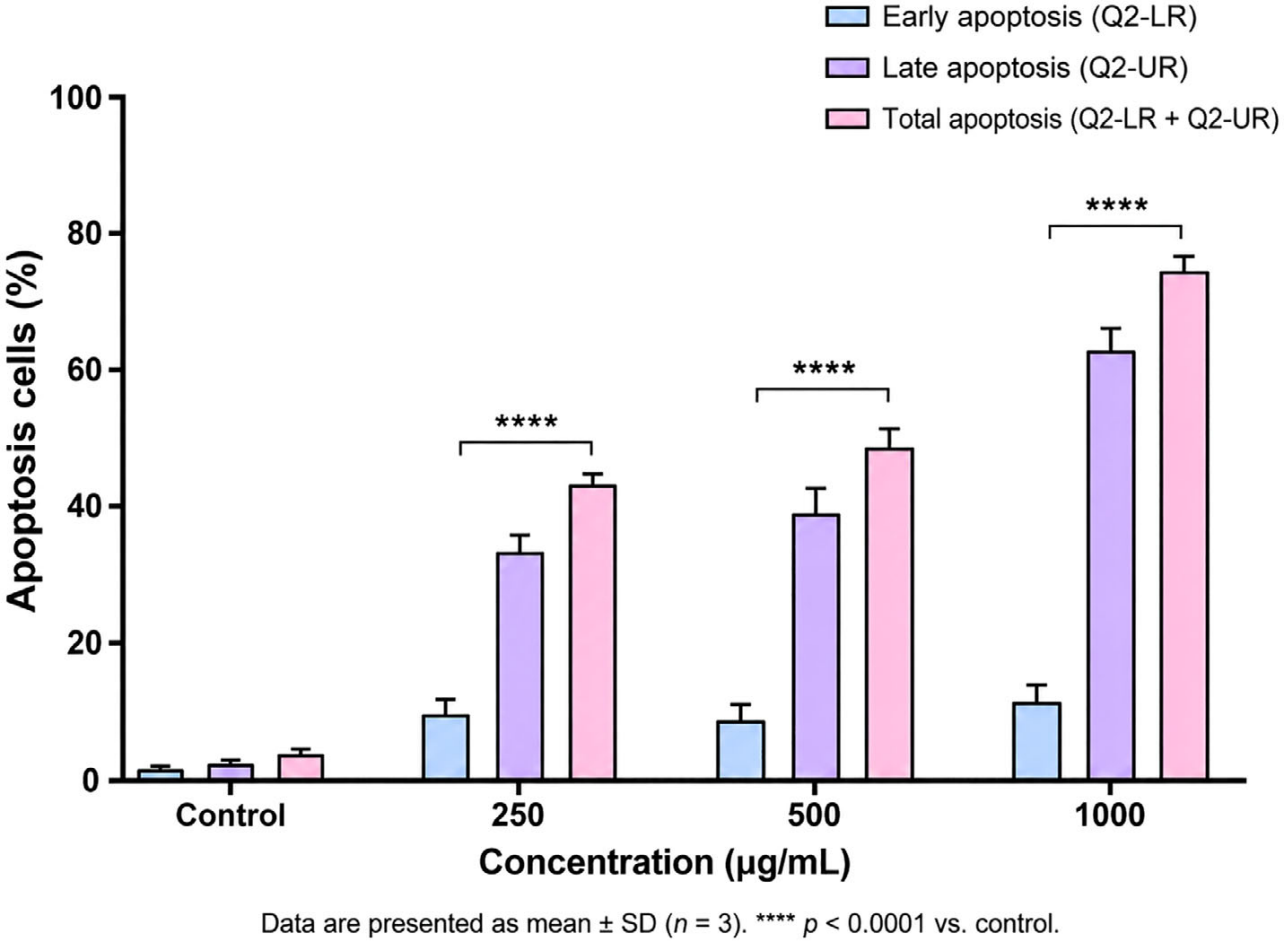
Quantification of HERAT‐induced apoptosis in PC‐3 (RRID:CVCL_0035) cells.

As shown in Figures 2 and 3, HERAT induced a marked and concentration‐dependent increase in apoptosis in PC‐3 cells (*p* < 0.0001). At 250 µg/mL, the total apoptotic population reached approximately 40%, while treatment with 500 µg/mL increased apoptosis to about 65%. At the highest concentration tested (1000 µg/mL), total apoptosis approached 85%, indicating a pronounced pro‐apoptotic effect.

These results demonstrate that HERAT effectively triggers programmed cell death in PC‐3 cells (RRID: CVCL_0035) in a dose‐dependent manner. The strong apoptotic response observed at higher concentrations suggests that growth inhibition induced by HERAT is predominantly mediated through apoptosis rather than nonspecific cytotoxicity, justifying further investigation of its underlying molecular mechanisms (Figures 2 and 3, Table S1).

Apoptosis induction in PC‐3 cells following treatment with HERAT was evaluated by flow cytometry. Representative dot plots illustrate the distribution of cell populations according to Annexin V–FITC and propidium iodide (PI) staining. Cells were treated with increasing concentrations of HERAT (250, 500, and 1000 µg/mL) for 24 h before staining and analysis. Quadrants Q2‐LL, Q2‐LR, Q2‐UR, and Q2‐UL correspond to viable (Annexin V^−^/PI^−^), early apoptotic (Annexin V^+^/PI^−^), late apoptotic (Annexin V^+^/PI^+^), and necrotic (Annexin V^−^/PI^+^) cell populations, respectively.

The percentage of apoptotic PC‐3 (RRID:CVCL_0035) cells following HERAT treatment was quantified using Annexin V–FITC/propidium iodide staining. Data are presented as the mean ± standard deviation (SD) of three independent experiments (*n* = 3). Statistical significance was determined using two‐way analysis of variance (ANOVA) followed by Bonferroni's post hoc test. ****p < 0.0001 compared with the control group.

#### 2.2.3 Cell Cycle Modulation

The effect of HERAT on cell‐cycle progression was evaluated in PC‐3 (RRID:CVCL_0035) cells by flow cytometric analysis. Treatment with HERAT resulted in a pronounced disruption of cell‐cycle distribution, reflecting its antiproliferative and mechanistic anticancer effects.

**FIGURE 4:**
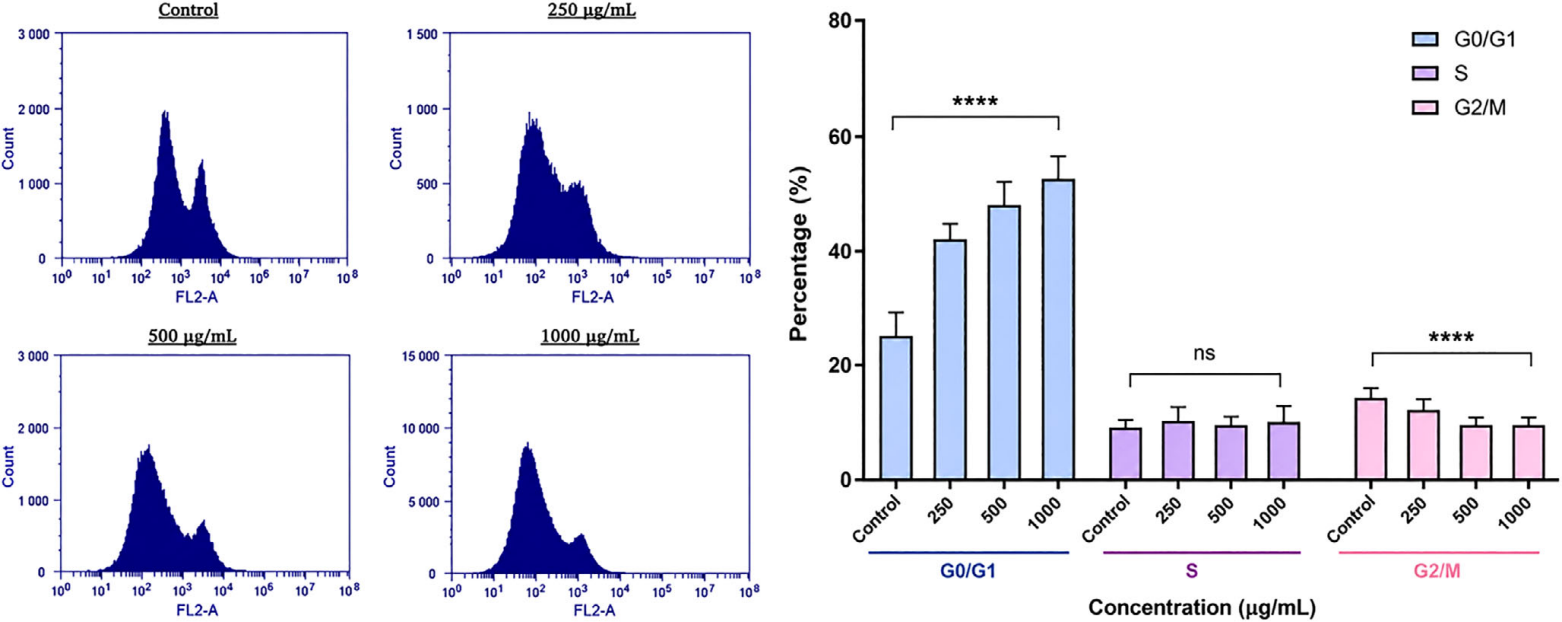
Effect of HERAT on cell‐cycle progression in PC‐3 (RRID:CVCL_0035) cells.

HERAT treatment induced a significant accumulation of cells in the G_0_/G_1_ phase, indicating an arrest at this checkpoint and thereby preventing progression into DNA synthesis and subsequent cell division. At the highest concentration tested (1000 µg/mL), approximately 60% of the cell population was retained in the G_0_/G_1_ phase (Figure 4 and Table S1). Concurrently, a marked reduction in the proportion of cells in the S phase was observed, suggesting effective inhibition of DNA replication.

A slight increase in the G_2_/M phase population was also detected following HERAT treatment, which may reflect differential regulation of cell‐cycle checkpoints or a compensatory cellular response to DNA damage. Overall, these findings demonstrate that HERAT exerts a dose‐dependent modulatory effect on cell‐cycle progression, predominantly through G_0_/G_1_ arrest and suppression of S‐phase entry, thereby contributing to its observed antiproliferative activity.

(A) Representative DNA‐content histograms showing the distribution of PC‐3 cells in the G0/G1, S, and G2/M phases following treatment with HERAT (250, 500, and 1000 µg/mL) for 24 h, as determined by flow cytometry after propidium iodide (PI) staining. All experiments were performed in triplicate to ensure reproducibility.

(B) Quantitative analysis of the percentage of cells in the G0/G1, S, and G2/M phases. Data are presented as mean ± standard deviation (SD) of three independent experiments (*n* = 3). Statistical significance was assessed using two‐way analysis of variance (ANOVA) followed by Bonferroni's post hoc test. ****p < 0.0001 compared with the control group.

#### 2.2.4 Anticancer Effects of Identified Metabolites in HERAT

HERAT exhibited moderate antiproliferative activity against PC‐3 and MDA‐MB‐231 cancer cells, accompanied by significant induction of apoptosis and G0/G1 cell‐cycle arrest. LC–HRMS/MS analysis revealed a phytochemical profile dominated by flavonoids, anthraquinones, and phenolic amides, suggesting that the observed biological effects may result from the combined or synergistic action of multiple metabolites rather than a single active constituent.

Among the identified compounds, **luteolin (18.51%)** may represent one of the major contributors to the anticancer activity of HERAT. Previous studies have reported that luteolin inhibits cancer cell proliferation through modulation of cyclins and cyclin‐dependent kinases, resulting in cell‐cycle arrest and suppression of tumor growth [25]. In addition, luteolin promotes apoptosis by activating caspase cascades, increasing the expression of pro‐apoptotic proteins such as BAX, and reducing the expression of anti‐apoptotic proteins including BCL‐2 and BCL‐xL [26]. Furthermore, luteolin has been shown to inhibit angiogenesis and suppress cancer cell migration and invasion through modulation of several signaling pathways, including PI3K/AKT/mTOR, STAT3, and Wnt/*β*‐catenin [27]. These pathways are also closely associated with cell survival, proliferation, and autophagy regulation [28]. Therefore, the relatively high abundance of luteolin in HERAT suggests it may substantially contribute to the antiproliferative and pro‐apoptotic effects observed in the present study.

Another major constituent, **luteolin‐7‐*O*‐*β*‐D‐glucoside (11.86%)**, may also contribute to the biological activity of the extract. Although glycosylation can modify the pharmacokinetic properties of flavonoids, luteolin‐7‐*O*‐*β*‐D‐glucoside retains biological activities similar to those of its aglycone precursor. Previous studies have demonstrated that this compound inhibits cancer cell migration and invasion through suppression of matrix metalloproteinases and modulation of ERK signaling pathways [29]. Moreover, it has been reported to induce cell‐cycle arrest and promote apoptotic cell death in different cancer models [30, 31]. The coexistence of luteolin and luteolin‐7‐*O*‐*β*‐D‐glucoside may therefore generate complementary or synergistic effects that enhance the overall anticancer potential of HERAT.

**Subaphyllin (feruloylputrescine, 14.04%)**, a hydroxycinnamic acid derivative, may also participate in the antiproliferative activity of HERAT. Hydroxycinnamic acid derivatives and related phenolic amides have been associated with modulation of signaling pathways involved in cellular stress responses, apoptosis, and cell‐cycle regulation [32, 33, 34]. Several studies have reported that compounds in this class may interfere with kinase signaling, induce DNA damage responses, and trigger apoptosis in cancer cells [35]. Although the specific anticancer activity of subaphyllin remains less extensively characterized than that of flavonoids, its relatively high abundance suggests that it may contribute, at least in part, to the growth inhibition and cell‐cycle alterations observed in the present study.

**Emodin (10.45%)** is another major metabolite that may play an important role in the biological activity of HERAT. Previous studies have shown that emodin inhibits cancer cell proliferation through multiple mechanisms, including reactive oxygen species generation, mitochondrial dysfunction, and activation of caspase‐dependent apoptotic pathways [36, 37]. Moreover, emodin has been reported to induce cell‐cycle arrest, thereby limiting cancer cell division and survival [38]. It can also suppress cancer cell migration and invasion through regulation of matrix metalloproteinases [39]. These effects are associated with the inhibition of major oncogenic signaling cascades, modulation of PI3K/AKT/mTOR and MAPK/ERK signaling [40]. Collectively, these findings suggest that emodin may contribute to the apoptosis induction and cell‐cycle arrest observed in PC‐3 and MDA‐MB‐231 cells.

**Feruloyltyramine (8.20%)** may further reinforce the anticancer potential of the extract. Beyond its antioxidant properties, this phenolic amide has been associated with inhibition of cellular adhesion processes involved in tumor progression and metastasis, particularly through modulation of P‐selectin‐related pathways [41]. In addition, studies have suggested that feruloyltyramine exhibits selective cytotoxicity toward cancer cells while showing lower toxicity toward normal cells [42]. Consequently, this metabolite may contribute not only to growth inhibition but also to the reduction of cancer cell migration and metastatic potential.

Taken together, the biological effects observed for HERAT may be attributed to the combined action of **luteolin, luteolin‐7‐*O*‐*β*‐D‐glucoside, emodin, subaphyllin, and feruloyltyramine**. These metabolites have been reported to modulate multiple molecular pathways involved in cell proliferation, apoptosis, cell‐cycle progression, oxidative stress responses, and potentially autophagy regulation, including PI3K/AKT/mTOR, MAPK/ERK, BCL‐2 family proteins, and cyclin‐dependent kinases [25, 26, 27, 28, 29, 30, 31, 32, 33, 34, 35, 36, 37, 38, 39, 40, 41, 42]. The simultaneous modulation of these targets may explain the significant G0/G1 arrest and apoptotic effects observed in the present study.

Although the present investigation was limited to PC‐3 and MDA‐MB‐231 cells, several of the major metabolites identified in HERAT have demonstrated antiproliferative, pro‐apoptotic, and cell‐cycle‐modulating activities in a broad range of malignancies, including colorectal, liver, lung, cervical, and hematological cancers [25, 26, 27, 28, 29, 30, 31, 32, 33, 34, 35, 36, 37, 38, 39, 40, 41, 42]. These observations suggest that similar biological effects may potentially occur in other cancer cell lines. Nevertheless, substantial molecular heterogeneity exists among tumor types, and cellular responses may vary according to genetic alterations, receptor expression profiles, and activation of signaling pathways. Therefore, additional studies involving other cancer models and non‐tumoral cell lines are warranted to evaluate the broader applicability, selectivity, and molecular mechanisms of HERAT as a potential anticancer agent.

#### 2.2.5 In Silico Studies

To support and rationalize the in vitro anticancer effects observed for HERAT, in silico studies were performed to explore the potential molecular interactions and biological targets of its major constituents. Computational analyses, including molecular docking, provide valuable insight into structure–activity relationships and possible mechanisms of action, thereby bridging experimental findings with molecular‐level predictions [43, 44, 45]. This integrative approach strengthens the interpretation of biological data and provides a scientific basis for understanding the multitarget anticancer potential of HERAT [24, 46].

#### 2.2.6 ADME‐Toxicity Profiles

To further support the pharmacological relevance of the major metabolites identified in HERAT, an integrated ADME–toxicity–drug‐likeness assessment was performed for the five principal compounds: luteolin, luteolin‐7‐*O*‐*β*‐D‐glucoside, emodin, feruloyltyramine, and subaphyllin. These structurally diverse phenolic and anthraquinone derivatives are likely to act cooperatively, contributing to the extract's observed antiproliferative, pro‐apoptotic, and ROS‐modulating activities in PC3 and MDA‐MB‐231 cells.

Table 3 summarizes and compares their physicochemical characteristics, absorption and distribution tendencies, metabolic interactions, predicted excretion parameters, and toxicity profiles, providing a comprehensive pharmacokinetic and safety overview of the major constituents.

**TABLE 3:** Integrated ADME and toxicity profiles of the major phytochemicals identified in HERAT.

Category	Parameter	Luteolin	Luteolin‐7‐*O*‐β‐D‐glucoside	Emodin	Feruloyltyramine	Subaphyllin
Physicochemical properties	Molecular Weight (g/mol)	286.24	448.38	270.24	313.35	264.32
	Log *P*	2.24	0.90	3.81	1.99	0.04
	TPSA (Å^2^)	111.13	190.28	94.83	78.79	84.58
Absorption	GI Absorption	High	Low	High	High	High
	P‐gp Substrate	No	**Yes**	No	No	No
	Water Solubility (log *S*)	−3.71	**−3.65**	−3.67	−3.03	−1.70
	Caco‐2 cell permeability (nm/s)	−5.19	−6.33	−5.14	−5.06	−5.81
	Human intestinal absorption (HIA%)	No	Yes	No	No	Yes
						
Distribution	PPB (%)	97.6	81.1	95.2	92.2	50.6
	BBB Penetration	No	No	No	No	No
	Skin Permeation (Log *K*p, cm/s)	−6.25	−8.00	−6.02	−6.72	−7.18
Metabolism	CYP Inhibition	1A2, 3A4 2D6	None	1A2, 3A4	2D6 3A4	None
Excretion	CL_(plasma)_ (mL/min/Kg)	8.48	3.65	2.93	11.27	7.90
	Predicted T^1/2^ (h)	1.37	3.84	1.43	1.12	1.00
						
Toxicity	Hepatotoxicity	No	No	No	No	No
	hERG Inhibition	0.069	0.019	0.037	0.475	0.356
	DILI	0.796	0.984	0.826	0.172	0.121
	AMES toxicity	0.65	0.92	0.91	0.39	0.54
	Carcinogenicity	0.689	0.342	0.769	0.275	0.215
	Cytotoxicity (A549)	0.669	0.673	0.905	0.278	0.234
	Skin sensitization	0.929	0.998	0.94	0.928	0.979
	Oral rat acute toxicity LD_50_ (mg/kg)	0.51	0.046	0.49	0.078	0.12
	Toxicity Class	5	5	**5**	4	4
Drug likeness	Lipinski Compliance	Yes	**No**	Yes	Yes	Yes
	Bioavailability Score	**0.55**	**0.17**	**0.55**	**0.55**	**0.55**

*Note*: Values shown as mean predictions. Log P, consensus log P (SwissADME) ; PPB, plasma protein binding; DILI, drug‐induced liver injury probability; GI Absorp., gastrointestinal absorption; BBB Perm., blood‐brain barrier permeation.

Most compounds exhibited physicochemical profiles compatible with oral drug candidates, with molecular weights < 500 g/mol (except the glucoside) and TPSA values within the range supporting good permeability. Luteolin, emodin, feruloyltyramine, and subaphyllin showed acceptable Log *p* values (0.04–3.81) and TPSA < 120 Å^2^, parameters associated with balanced lipophilicity and efficient cell membrane passage. These trends are consistent with the high or moderate predicted GI absorption for four out of the five molecules. Luteolin‐7‐*O*‐glucoside showed low predicted absorption due to its large TPSA (190 Å^2^), but this does not exclude activity inside cells, considering its possible deglycosylation by gut microbiota or intracellular β‐glucosidases, an established mechanism improving bioavailability of glycosylated flavonoids [47, 48, 49]. The SwissADME bioavailability radar (Figure 7) provides a visual summary of the key physicochemical parameters governing oral suitability, further illustrating the favorable balance of size, polarity, solubility, flexibility, and lipophilicity for the aglycone compounds. In addition, Feruloyltyramine, emodin, and luteolin exhibited high plasma protein binding (PPB 92–97%), a common feature of polyphenolic anticancer agents, which often prolongs systemic exposure and enhances tissue distribution [48, 50]. Subaphyllin displayed moderate PPB (50.6%), suggesting a potentially higher free fraction. None of the molecules are predicted to cross the BBB, aligning with the intended peripheral anticancer application and reducing concerns regarding CNS toxicity [51].

Luteolin, emodin, and feruloyltyramine were predicted to inhibit several major CYP isoforms, notably CYP1A2, CYP3A4, and CYP2D6, in agreement with experimental reports showing that flavonoids and anthraquinones frequently modulate phase I metabolism [52]. Luteolin is known to inhibit CYP3A4‐mediated pathways [53], while emodin has been shown to interfere with CYP3A4 and hepatocyte metabolic activity [54]. Such moderate CYP inhibition is typical for polyphenolic scaffolds and may even prolong in vivo activity by reducing metabolic clearance [55]. In contrast, luteolin‐7‐*O‐β*‐D‐glucoside and subaphyllin exhibited a narrower CYP inhibition profile. Their limited CYP engagement suggests a more favorable metabolic safety profile and a lower risk of drug–drug interactions.

Predicted clearance values indicate slow to moderate elimination, with T½ ranging from 1.0 to 3.84 h, compatible with short‐acting anticancer phytochemicals [50]. The glucoside form showed the longest half‐life, consistent with its hydrophilic nature and lower membrane diffusion.

The toxicity profile of the compounds was highly favorable, indicating a promising safety margin for development. No hepatotoxicity was predicted for any compound, and the risk of cardiotoxicity was low, as evidenced by minimal hERG inhibition probabilities, particularly for luteolin, emodin, and their glucosides. The predicted AMES mutagenicity probabilities for the five major HERAT constituents ranged between 0.39 and 0.92, indicating an overall low to moderate mutagenic potential. These values reflect the known behavior of polyphenols and anthraquinones, which may trigger in silico mutagenicity alerts due to their planar aromatic structures, despite often demonstrating safe profiles in vivo [48]. Generally, the AMES predictions suggest that the compounds do not pose a high mutagenic risk. Furthermore, the predicted rat acute oral toxicity (LD_50_) probabilities for the five major compounds studied ranged from 0.046 to 0.51, indicating an overall low to moderate acute‐toxicity profile. This positive profile is solidified by the classification of all compounds into low acute toxicity categories (4 to 5).

**FIGURE 5:**
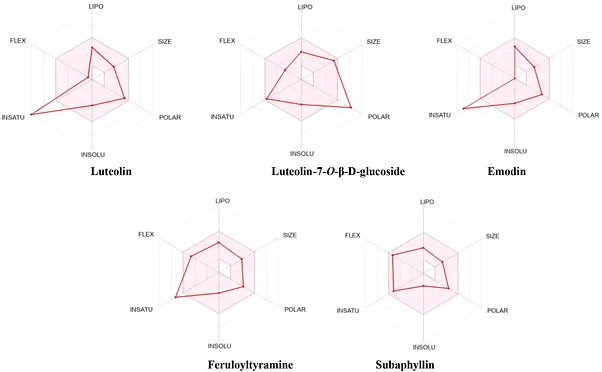
SwissADME bioavailability radar plots of the five major phytochemicals of HERAT, illustrating lipophilicity, size, polarity, solubility, saturation, and flexibility parameters.

According to Lipinski's rule of five, four of the five investigated compounds fulfilled all drug‐likeness criteria, except for luteolin‐7‐*O*‐*β‐*D‐glucoside, whose elevated molecular weight and large topological polar surface area (TPSA) exceeded acceptable thresholds for passive oral absorption. Within the series, luteolin and emodin stood out as the most promising drug‐like candidates, combining balanced lipophilicity, favorable absorption parameters, and a consistently benign safety profile. Their physicochemical properties fall within the optimal range for oral bioavailability, supporting their potential as orally active anticancer leads. The SwissADME bioavailability radar (Figure 5) further illustrates the overall suitability of these compounds, providing a visual representation of key parameters governing oral drug performance and bioactivity.

#### 2.2.7 Target Prediction Analysis

The in silico target prediction analysis of the five major phytochemicals, feruloyltyramine, subaphyllin, emodin, luteolin‐7‐*O*‐β‐D‐glucoside, and luteolin, revealed distinct yet complementary polypharmacological profiles that align with their reported biological activities. These findings support the hypothesis that the anticancer potential of *A. tenuifolius* roots arises from synergistic multi‐target interactions rather than single‐target specificity, a hallmark of many bioactive natural products [56, 57]. Aglycone compounds such as luteolin and emodin demonstrated strong predicted affinities toward kinase‐related targets. This is consistent with the established role of flavonoids and anthraquinones in modulating protein kinases that regulate cell proliferation and survival pathways. Such interactions highlight their potential to disrupt oncogenic signaling cascades [58]. Notably, feruloyltyramine exhibited predicted interactions with proteases and oxidoreductases, consistent with literature describing its antioxidant, anti‐inflammatory, and enzyme‐modulating properties [59, 60]. This broader target spectrum suggests that feruloyltyramine contributes to redox balance and inflammatory regulation, complementing the kinase‐focused activity of other metabolites.

In contrast, the glycosylated metabolite luteolin‐7‐*O*‐β‐D‐glucoside demonstrated a markedly reduced affinity for kinase‐related targets and a broader distribution across other enzyme classes. This shift reflects the well‐established impact of glycosylation on the pharmacokinetic and pharmacodynamic behavior of flavonoids, whereby conjugation with sugar residues modulates membrane permeability, intracellular transport, and target engagement [61, 62]. These findings highlight a critical structure–activity relationship: while luteolin itself shows strong kinase‐centered interactions; its glucoside derivative adopts a more diverse but less kinase‐focused profile.

The “Other Enzymes” category included predicted targets such as phosphatases, epigenetic regulators, ROS‐associated oxidoreductases, and DNA‐processing enzymes. These interactions suggest additional mechanisms through which the metabolites may exert cytotoxic and antiproliferative effects. Importantly, these predictions align with experimental evidence of ROS‐mediated apoptosis and cell‐cycle arrest [63, 64].

**FIGURE 6:**
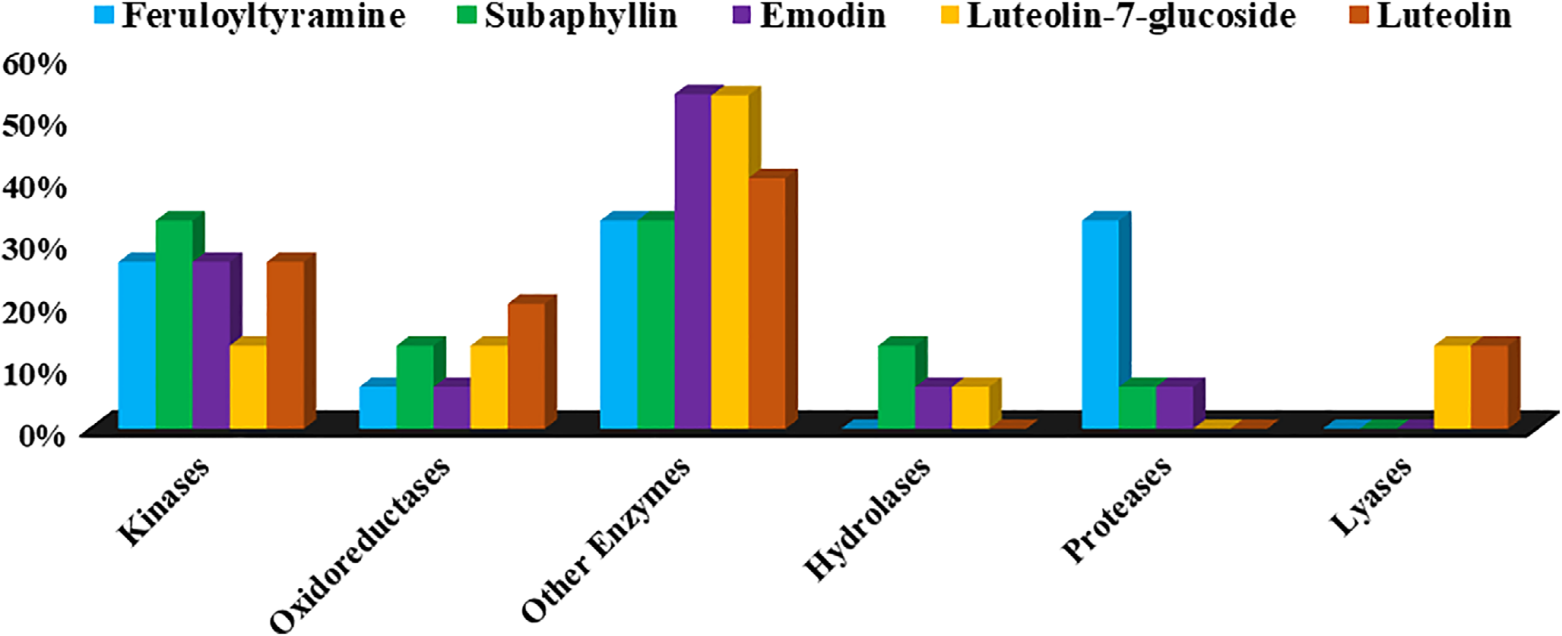
Target prediction profiles and probability scores for the five principal phytoconstituents of HERAT.

Taken together, integrating kinase‐centered interactions with additional enzyme classes underscores the multifaceted pharmacological landscape of *A. tenuifolius* root metabolites. This layered targeting strategy may account for the extract's ability to disrupt proliferative signaling simultaneously, induce oxidative stress, and compromise DNA integrity—hallmarks of effective anticancer agents. The resulting multitarget profile is consistent with the in vitro evidence of cytotoxicity, pro‐apoptotic activity, and cell‐cycle modulation observed for the root extract (HERAT), thereby providing a mechanistic foundation for its multitarget pharmacological behavior. The convergence between in silico predictions and experimental findings highlights the robustness of computational target profiling as a complementary tool for mechanistic exploration. By revealing diverse enzyme classes beyond kinases, the analysis provides a framework for future validation studies aimed at dissecting the precise molecular pathways underlying the anticancer activity of *A. tenuifolius*. These insights reinforce the broader principle that natural products achieve therapeutic efficacy through coordinated, multi‐target interactions across complex biological networks. Figure 6 summarizes the predicted biological targets and associated probabilities for the five major compounds, illustrating their most relevant activity profiles. Beyond the five major enzyme classes analyzed, the Other Enzymes category included specialized targets such as phosphatases, transferases, epigenetic modifiers, and DNA‐associated enzymes, highlighting additional mechanisms by which these compounds may exert their biological effects.

#### 2.2.8 Molecular Docking Analysis

To assess whether the metabolite profile identified in *A. tenuifolius* roots underlies the apoptosis‐dominant phenotype observed in vitro, a phenotype‐driven docking strategy was employed. Rather than treating docking as a series of isolated binding simulations, this approach served as a mechanistic validation framework integrating LC–HRMS/MS‐based phytochemical profiling with the experimentally observed cell cycle arrest and apoptosis induction. In this context, the analysis extended beyond single‐target interactions to evaluate whether the major identified metabolites could collectively modulate multiple oncogenic pathways converging on key regulators of cancer cell proliferation and survival.

Target selection was guided by experimental findings: G0/G1 phase arrest directed attention to CDK2, while pronounced apoptosis induction supported the inclusion of BCL‐2. Additionally, the use of aggressive breast and prostate cancer models warranted the incorporation of AKT1, along with hormone receptor signaling pathways (ERα and AR). Binding energies (kcal/mol) are summarized in Table 4.

**TABLE 4:** Binding energy (kcal/mol) of major metabolites from *A. tenuifolius* Cav. roots extract (HERAT) against selected cancer‐related protein targets.

Protein target (PDB ID)	Reference ligand	Binding energy (kcal/mol)	Compound	Score (kcal/mol)
ERα—estrogen receptor (3ERT)	**OHT (Tamoxifen derivative)**	**−9.8**	Luteolin	**−8.3**
			Luteolin‐7‐*O*‐glucoside	−7.8
			Emodin	**−8.3**
			Feruloyltyramine	−8.1
			Subaphyllin	−6.2
AKT1 receptor (3O96)	**IQO**	**−14.4**	Luteolin	**−9.8**
			**Luteolin‐7‐*O*‐glucoside**	**−10.4**
			**Emodin**	**−10.3**
			Feruloyltyramine	−8.9
			Subaphyllin	−7.4
AR (2Q7I)	**Testosterone**	**−11.0**	Luteolin	**−8.9**
			Luteolin‐7‐*O*‐glucoside	**+2.6**
			Emodin	**−8.6**
			Feruloyltyramine	−5.0
			Subaphyllin	−6.7
CDK2 (1PYE)	**PM‐1**	**−9.8**	**Luteolin**	**−8.4**
			**Luteolin‐7‐*O*‐glucoside**	**−8.7**
			**Emodin**	**−9.0**
			**Feruloyltyramine**	**−8.0**
			Subaphyllin	−6.6
BCL‐2 (6O0K)	**Venetoclax**	**−12.2**	Luteolin	**−7.2**
			Luteolin‐7‐*O*‐glucoside	**−7.8**
			Emodin	**−7.4**
			Feruloyltyramine	−7.3
			Subaphyllin	−5.8

A binding affinity more negative than −7.0 kcal/mol is generally regarded as indicative of significant binding potential [46]. Consistent with the in silico target prediction analysis, the major constituents of HERAT—particularly luteolin, luteolin‐7‐*O*‐glucoside, emodin, and feruloyltyramine—exhibited strong binding affinities across multiple oncogenic targets. This observation reinforces the polypharmacological profile previously described, wherein these metabolites were predicted to modulate kinases, proteases, oxidoreductases, and other regulatory enzymes [65].

**FIGURE 7:**
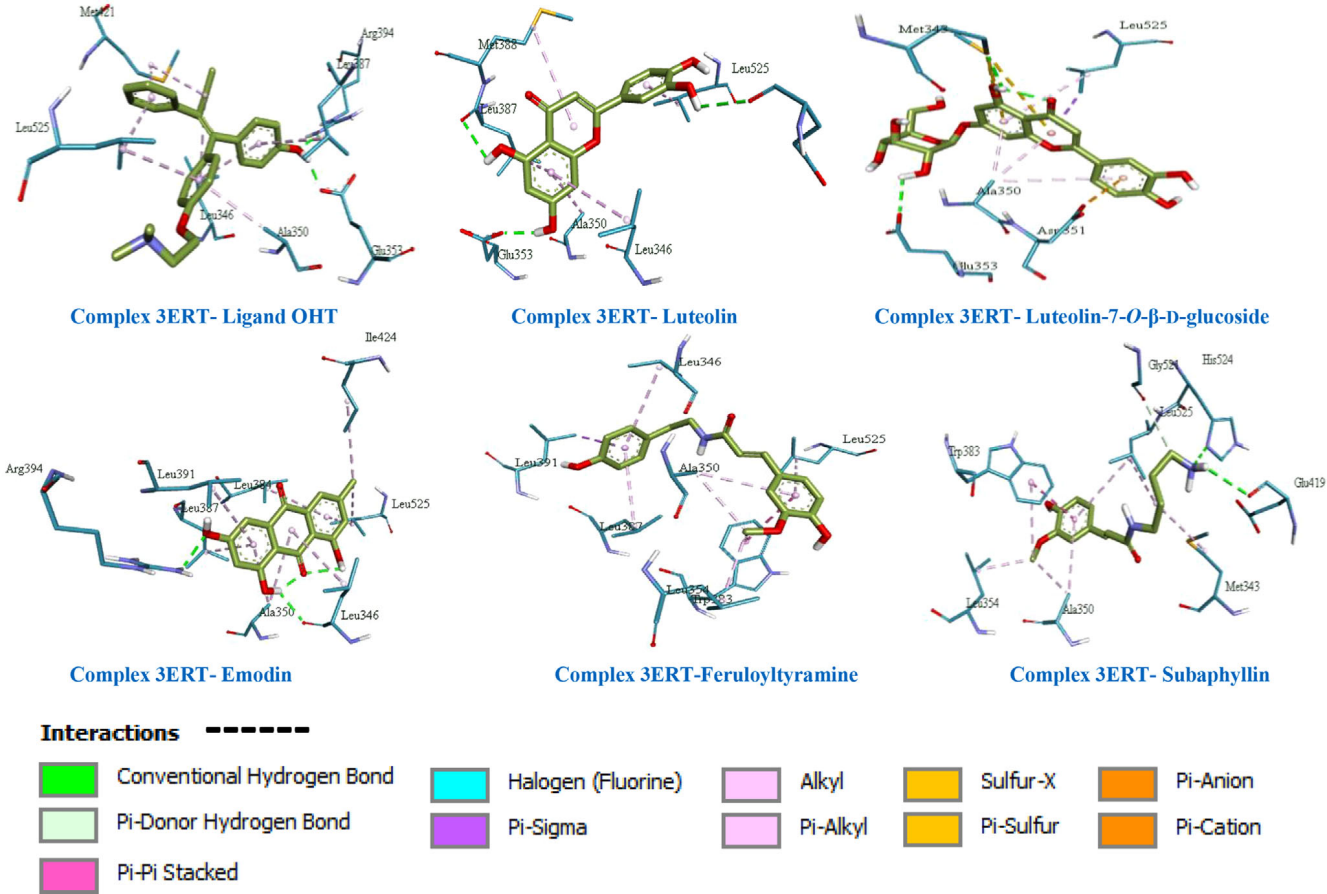
The best obtained poses of docking results with 3ERT and the different studied ligands with OHT as a positive control. Hydrogen bonds are represented as a green dashed line.

Luteolin and emodin demonstrated consistent binding to ERα (−8.3 kcal/mol), occupying the ligand‐binding domain in a manner comparable to 4‐hydroxytamoxifen, forming canonical hydrogen bonds with Glu353 and Arg394 (Figure 7).

**FIGURE 8:**
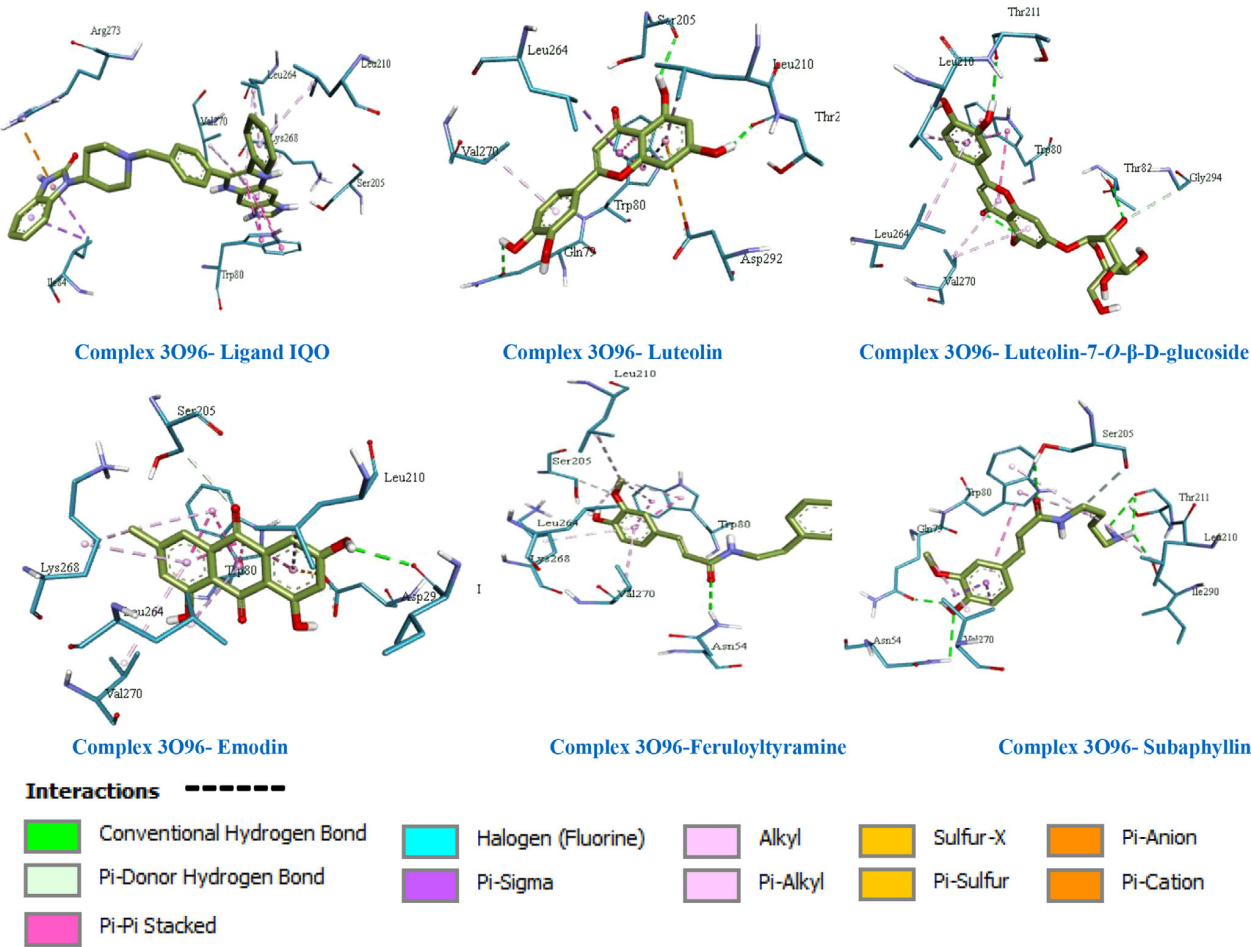
The best obtained poses of docking results with 3096 and the different studied ligands, with IQO as a positive control. Hydrogen bonds are represented as a green dashed line.

**FIGURE 9:**
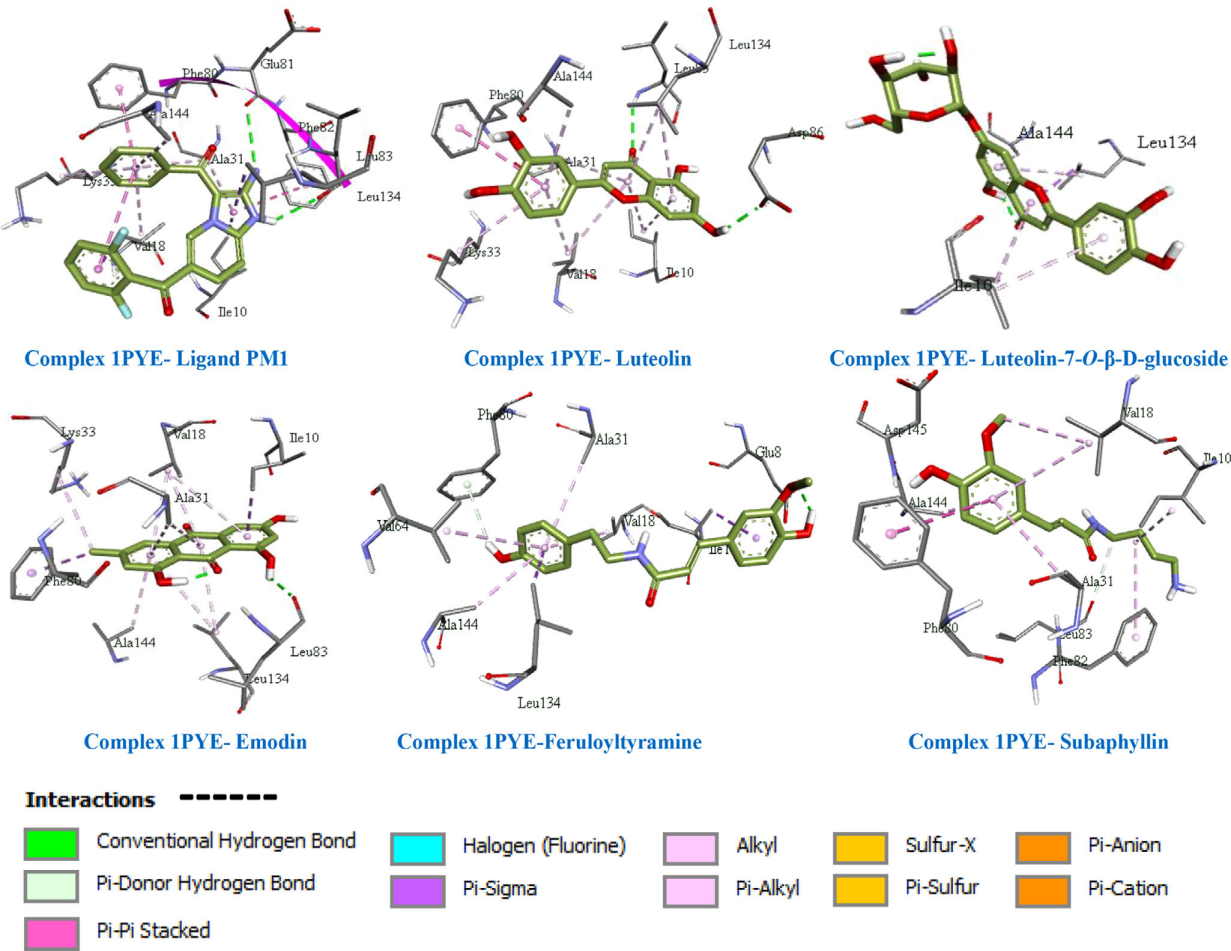
The best obtained poses of docking results with 1PYE and the different studied ligands, with PM1 as a positive control. Hydrogen bonds are represented as a green dashed line.

AKT1 exhibited the strongest predicted affinities (up to −10.4 kcal/mol), with ligands penetrating the ATP‐binding cleft and engaging catalytic residues, forming hydrogen bonds with Thr82, Thr211, Ser205, Ile290, and Gly294, stabilized by π–π stacking with Trp80 and hydrophobic contacts with Leu264 and Val270 (Figure 8). Given AKT1's central role as a survival hub linking PI3K activation to downstream anti‐apoptotic signaling, this interaction provides a structural explanation for the observed apoptosis induction and G0/G1 arrest. CDK2 docking supports ATP‐site occupation via conserved hinge interactions (Glu81, Leu83) (Figure 11), consistent with suppression of the G1/S transition. Emodin, luteolin, luteolin‐7‐*O*‐glucoside, and feruloyltyramine exhibited favorable binding energies (−9.0, −8.4, −8.7, and −8.0 kcal/mol, respectively). All ligands occupied the ATP‐binding cleft, forming hydrogen bonds with Asp86, Glu81, and Leu83, and hydrophobic contacts with Phe80, Il10 and Val18 (Figure 9). These interactions resemble those of known CDK2 inhibitors and support the experimentally observed G0/G1 arrest [66].

**FIGURE 10:**
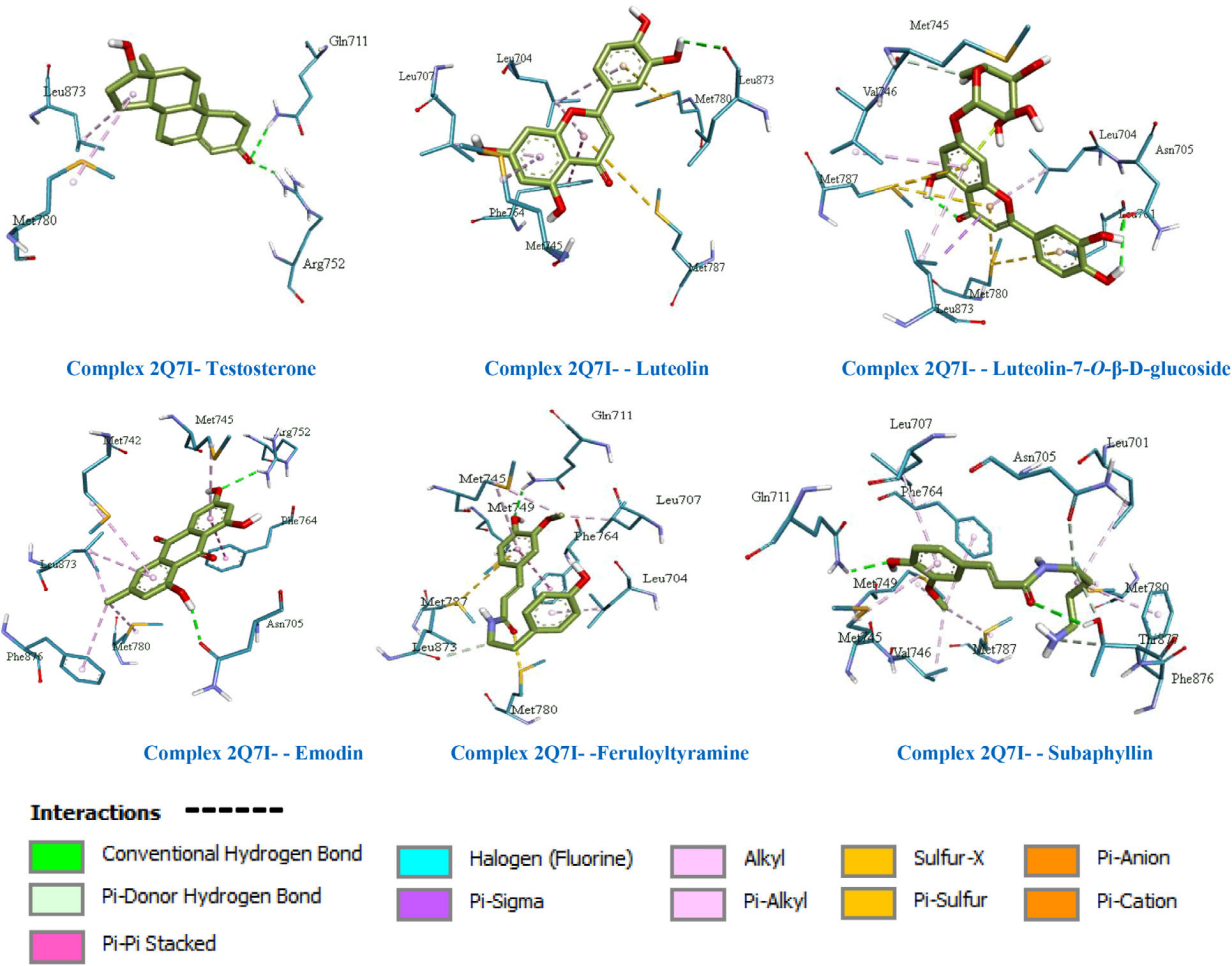
The best obtained poses of docking results with 2Q7I and the different studied ligands, with testosterone as a positive control. Hydrogen bonds are represented as a green dashed line.

Luteolin and emodin displayed favorable predicted affinities toward the androgen receptor (AR) ligand‐binding domain (−8.9 and −8.6 kcal/mol, respectively). Both compounds were accommodated within the hydrophobic cavity and formed hydrogen bonds with Arg752 and Asn705, as well as stabilizing hydrophobic contacts involving Leu704, Met742, and Phe764 (Figure 10). These interactions indicate that the planar aglycone scaffolds are structurally compatible with the AR binding pocket and may contribute to the modulation of androgen‐dependent signaling pathways relevant to prostate cancer progression [67]. In contrast, luteolin‐7‐*O*‐glucoside showed negligible binding (+2.6 kcal/mol), most likely due to steric hindrance introduced by the glycosidic moiety, which limits penetration into the relatively constrained receptor cavity. This observation highlights a structure–activity relationship in which glycosylation reduces accessibility to the AR ligand‐binding site.

**FIGURE 11:**
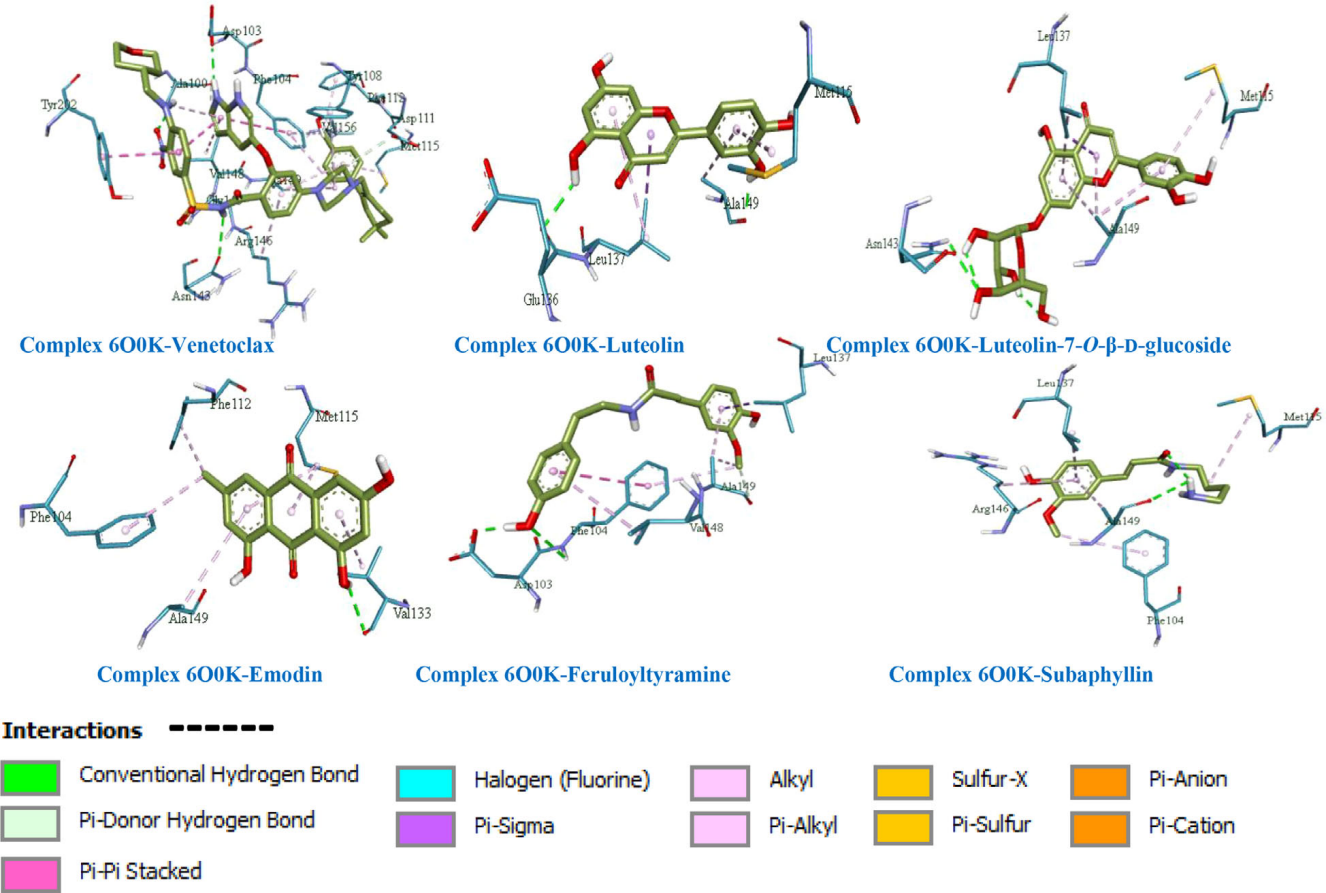
The best obtained poses of docking results with 6O0K and the different studied ligands, with venetoclax as a positive control. Hydrogen bonds are represented as a green dashed line.

The evaluated secondary metabolites exhibited moderate predicted binding affinities toward the BH3‐binding groove of BCL‐2 (PDB ID: 6O0K), with docking energies ranging from approximately −6.0 to −7.8 kcal/mol. The ligands established stabilizing contacts with residues such as Tyr108, Asp111, Asn143, Arg146, and Glu136 (Figure 11), indicating partial occupancy of the hydrophobic groove responsible for sequestration of pro‐apoptotic proteins. Although the predicted affinities are weaker than those reported for clinically optimized BH3 antagonists such as Venetoclax, these interactions suggest that polyphenolic scaffolds may contribute to modulation of mitochondrial apoptosis signaling in a complementary manner rather than through high‐affinity inhibition [68].

Importantly, although similar interactions have been reported for individual flavonoids, the relevance of the present findings lies in their integration into a multitarget framework involving AKT1, CDK2, and BCL‐2. This convergence of predicted interactions among survival kinases, nuclear receptors, and apoptosis regulators supports the hypothesis that the extract's biological activity arises from coordinated modulation of interconnected oncogenic pathways rather than from isolated receptor binding.

Collectively, the docking results delineate a coherent multi‐target mechanistic framework that mirrors the potent antiproliferative and pro‐apoptotic effects observed for HERAT in vitro. The complementary binding profiles of its major constituents across ERα, AKT1, AR, CDK2, and BCL‐2 provide a strong structural rationale for the extract's biological activity. This convergence of targets involved in cell‐cycle progression, survival signaling, and mitochondrial apoptosis supports a synergistic mode of action, offering a compelling explanation for the marked cytotoxicity and apoptosis induction measured experimentally.

## 3 Conclusions

This study provides the first comprehensive biological and computational investigation of the anticancer potential of *A. tenuifolius* roots, an organ previously unreported to possess such activity. The hydroethanolic root extract (HERAT) exhibited pronounced antiproliferative effects, inducing significant G0/G1 cell‐cycle arrest and robust apoptosis, with up to 85% of PC‐3 cells undergoing programmed cell death at the highest tested concentration. LC–HRMS/MS profiling revealed a chemically diverse metabolite composition dominated by flavonoids and phenolic derivatives, particularly luteolin, luteolin‐7‐*O*‐*β*‐D‐glucoside, emodin, feruloyltyramine, and subaphyllin. This root‐specific phytochemical profile differs markedly from that previously reported for the aerial parts of *A. tenuifolius*, which were characterized mainly by adenosine, chlorogenic acid, aloesin, and hydroxysafflor Yellow A and displayed limited or no cytotoxic activity. The substantial differences observed in both phytochemical composition and biological activity between the two plant organs underscore the organ‐dependent distribution of bioactive metabolites and highlight the importance of organ‐specific metabolomic investigations in natural product research. Furthermore, the integrated in silico analyses provided a coherent mechanistic explanation for the observed in vitro effects, demonstrating that the major metabolites are capable of multitarget modulation of ERα, AKT1, CDK2, AR, and BCL‐2, key regulators of proliferation, survival signaling, cell‐cycle progression, and apoptosis. Strong binding affinities together with favorable ADME–Tox profiles further support the potential of these compounds as safe and drug‐like multitarget anticancer candidates. Collectively, these findings identify *A. tenuifolius* roots as a promising source of bioactive metabolites with multitarget anticancer potential and establish a solid scientific basis for future metabolite isolation, structure–activity relationship studies, and in vivo validation aimed at the development of novel anticancer lead compounds.

## 4 Experimental Section

### 4.1 Plant Material

*A. tenuifolius* Cav. was collected in March 2019 from Ghardaïa province (Algerian Septentrional Sahara) (Latitude: 32° 9′ 18″ N, Longitude: 4° 20′ 45″ E, Altitude: 476 m). Its identification was performed by Prof. Gérard de Belair (University Badji Mokhtar‐Annaba, Algeria), and it's listed in the herbarium of University Mentouri‐Constantine 1 under the Voucher code lostAst.03.19.

### 4.2 Extraction

The dried roots of *A. tenuifolius* (350 g) were finely ground and macerated in a hydroethanolic solution (ethanol/water, 80:20 v/v) for 3 × 24 h. The resulting crude extract was concentrated under reduced pressure until complete evaporation, yielding 4.5 g of hydroethanolic extract [69].

### 4.3 Sample Preparation

The hydroethanolic extract of the roots of *A. tenuifolius* (HERAT) was initially dissolved in dimethyl sulfoxide (DMSO, Sigma‐Aldrich) and then serially diluted with RPMI medium to ensure that the final DMSO concentration did not exceed 0.1%. To remove any insoluble residues, the suspension was centrifuged at 5000 × g for 10 min, and the resulting supernatant was subsequently passed through a 0.22 µm sterile syringe filter. The clarified filtrate was further diluted in RPMI medium to obtain the desired concentrations for biological assays.

### 4.4 LC‐HRMS/MS Analyses (Liquid Chromatography‐High Resolution Mass Spectrometry)

Prior to LC‐ESI‐DDA‐HRMS/MS analysis, the hydroethanolic root extract was reconstituted in ethanol, vortex‐mixed until complete dissolution, and centrifuged to remove any insoluble particles. The supernatant was subsequently filtered through a 0.22 µm syringe membrane filter and transferred into LC vials for analysis. LC‐ESI‐DDA‐HRMS/MS analysis was carried out using a Dionex Ultimate 3000 HPLC system integrated with a Thermo Scientific LTQ Orbitrap XL mass spectrometer. Chromatographic separation was conducted on a Waters BEH C18 column (1.7 µm, 2.1 × 150 mm) maintained at 45°C. The mobile phase was composed of solvent A (water with 0.1% formic acid) and solvent B (acetonitrile with 0.1% formic acid). A gradient program was employed, initiating at 5% of solvent B for the first 5 min, gradually increasing to 100% over the next 20 min, and held at that composition for an additional 7 min. The flow rate was maintained at 0.150 mL/min. MS/MS data were processed using MZmine 2.53, with detection thresholds set at 2E4 for positive mode and 2E3 for negative mode. Identification of the chemical constituents was achieved via the GNPS platform, facilitating comprehensive analysis of the phytochemical composition [70].

### 4.5 Cultivation of Cells

The MDA‐MB‐231 cell line, originally obtained from a pleural effusion of a mammary adenocarcinoma (ATCC HTB‐26; RRID: CVCL_0062), was cultured in DMEM (PAN Biotech, Aidenbach, Germany) supplemented with 10% fetal bovine serum (FBS; Dominique Dutscher, SA, Brumath, France) and 1% penicillin/streptomycin. Similarly, the PC‐3 cell line, originating from prostate cancer (ATCC CRL‐1435; RRID: CVCL_0035), was grown in RPMI 1640 medium (PAN Biotech, Aidenbach, Germany) containing 10% FBS and 1% penicillin/streptomycin. Both cell lines were maintained in a standard incubator at 37°C in a humidified atmosphere with 5% CO_2_ to ensure optimal growth conditions.

### 4.6 MTT Assay

The antiproliferative activity of HERAT was evaluated on the PC‐3 (ATCC CRL‐1435; RRID: CVCL_0035) and MDA‐MB‐231 (ATCC HTB‐26; RRID: CVCL_0062) cancer cell lines using the MTT assay, a well‐established method for assessing cell viability [71]. In this experiment, 5000 cells were seeded per well in 96‐well plates and exposed to HERAT at concentrations of 0.85, 2.54, 7.62, 22.86, 68.59, 205.8, 617.3, 1,851.8, 5,555.5, 16,666.6, and 50,000 µg/mL, Cells treated with culture medium containing 0.1% DMSO served as the negative control, whereas doxorubicin was used as the positive control. After 72 h of treatment, the medium was replaced with PBS, and 100 µL of MTT solution (5 mg/mL) was added to each well. The plates were incubated for an additional 2 h at 37°C in a humidified atmosphere with 5% CO_2_. Following incubation, the medium was carefully removed, and the resulting formazan crystals were dissolved in DMSO. Absorbance was measured at 570 nm using a microplate reader (Varioskan LUX, Thermo Scientific), providing a quantitative measure of cell viability. This method allowed the evaluation of the antiproliferative effects of the HERAT extract.

### 4.7 Apoptosis Assay

To assess apoptosis induced by HERAT, Annexin V‐FITC/PI staining was performed following the manufacturer's instructions (BD Pharmingen FITC Annexin V Apoptosis Detection Kit). PC‐3 (ATCC CRL‐1435; RRID: CVCL_0035) cells (10^5^ cells per well) were plated in 96‐well plates and treated with different concentrations of HERAT (250, 500, and 1000 µg/mL) for 24 h. After the treatment, cells were washed with PBS, centrifuged, and stained with 5 µL of Annexin V and 5 µL of propidium iodide (PI). The cells were incubated for 15 min at room temperature to allow proper staining. Following incubation, 400 µL of binding buffer was added to each sample, and flow cytometry analysis was performed. The stained cells were analyzed using the Accuri TM‐C6 flow cytometer (BD Biosciences), which provided valuable information on the apoptosis‐inducing effects of HERAT [72].

### 4.8 Evaluation of Cell Cycle Distribution

Cell cycle analysis was conducted using a cell cycle detection kit (BD Pharmingen PI/RNase staining buffer) to assess the effects of HERAT treatment on PC‐3 (ATCC CRL‐1435; RRID: CVCL_0035) cells. PC‐3 cells (10^6^ cells per well) were plated in 6‐well plates and exposed to HERAT at concentrations of 250, 500, and 1000 µg/mL for 24 h. Following treatment, the cells were collected, washed with cold PBS, and fixed in 70% ethanol at −20°C for 2 h to preserve cellular integrity. After fixation, the cells were centrifuged, and the pellet was resuspended in 0.5 mL of a staining solution containing propidium iodide and RNase. The samples were incubated at room temperature for 15 min to allow proper staining. The DNA content of the cells was then analyzed by flow cytometry using the Accuri TM‐C6 (BD Biosciences), providing valuable data on the distribution of the cell cycle phases after HERAT exposure.

### 4.9 Statistical Evaluation

All experiments were conducted in triplicate. Statistical analysis was performed using GraphPad PRISM software (version 8.0.2). Data are presented as the mean ± standard deviation (SD). A two‐way analysis of variance (ANOVA) was applied, followed by the Bonferroni post hoc test for multiple comparisons. Differences were considered statistically significant when the p‐value was less than 0.05.

### 4.10 In Silico Study

#### 4.10.1 ADME/Toxicity and Drug‐Likeness Prediction

The ADME properties of the major constituents of HERAT, luteolin, luteolin‐7‐*O*‐D‐glucoside, emodin, feruloyltyramine, and subaphyllin, were evaluated using the SwissADME web tool (http://www.swissadme.ch) and ADMETlab 3 (https://admetlab3.scbdd.com/server/evaluation). The assessment included molecular weight, lipophilicity (iLogP), number of hydrogen‐bond donors and acceptors, topological polar surface area (TPSA), aqueous solubility, gastrointestinal (GI) absorption, bioavailability score, and overall drug‐likeness according to multiple rule‐based filters (Lipinski, Ghose, Veber, Egan, and Muegge). In addition, predictions of P‐glycoprotein (P‐gp) substrate status and cytochrome P450 (CYP) enzyme inhibition profiles were generated for each compound.

Toxicity predictions were performed using ADMETlab 3, ProTox‐II (https://tox‐new.charite.de/protox_II/), and PkCSM (http://biosig.lab.uq.edu.au/pkcsm). ProTox‐II provided estimates of hepatotoxicity, cytotoxicity, mutagenicity, carcinogenicity, immunotoxicity, LD_50_ values, toxicity classes, and structural toxicity alerts. PkCSM was employed to predict hERG inhibition, hepatotoxicity, Ames mutagenicity, skin sensitization, and acute and chronic oral toxicity using graph‐based structural signatures. Outputs from all platforms were consolidated to generate an integrated toxicity profile for each compound.

#### 4.10.2 Target Prediction

Protein target prediction was performed using SwissTargetPrediction (http://www.swisstargetprediction.ch) based on 2D and 3D molecular similarity to known ligands. For each molecule, the top predicted human protein targets were extracted along with their probability scores. These targets were categorized into functional classes such as hormone receptors (ERα, AR), kinases (AKT1, CDKs, MAPKs), topoisomerases, and redox‐related enzymes. Only targets with the highest probabilities were selected for integration with docking studies.

#### 4.10.3 Molecular Docking

Five protein structures were selected for molecular docking based on their biological relevance to the in vitro assays: estrogen receptor α (ERα, PDB ID: 3ERT), cyclin‐dependent kinase 2 (CDK2, PDB ID: 1PYE), AKT1 kinase (PDB ID: 3O96), androgen receptor (AR, PDB ID: 2Q7I), and the anti‐apoptotic protein BCL‐2 (PDB ID: 6O0K). The crystal structures were retrieved from the Protein Data Bank (RCSB PDB) [73]. The selected structures correspond to well‐characterized complexes previously reported for ERα [74], CDK2 [75], AKT1 [76], AR [77], and BCL‐2 [78].

All target proteins (3ERT, 1PYE, 3O96, 2Q7I, and 6O0K) were prepared using PyMOL (Schrödinger, LCC, 2021) (https://pymol.org) in combination with the Drug Discovery plugin [79]. Cocrystallized ligands, ions, and non‐essential water molecules were removed, hydrogen atoms were added at physiological pH (7.4), missing residues were repaired where applicable, and the structures were energy‐minimized to relieve steric clashes. Gasteiger partial charges and AutoDock atom types were assigned automatically, and the prepared proteins were exported in PDBQT format [80]. The 2D chemical structures of the identified phytochemicals were retrieved from the PubChem database and converted into 3D conformations using Chem3D (PerkinElmer Informatics, Waltham, MA, USA). Geometry optimization and energy minimization were performed using the MMFF94 molecular mechanics force field [81] until stable conformations were obtained. Hydrogen atoms were added, Gasteiger charges were assigned, rotatable bonds were identified, and the optimized ligands were converted into PDBQT format for docking. Molecular Docking simulations were performed using AutoDock Vina [23]. Grid boxes were centered on the coordinates of the co‐crystallized ligands. Binding affinities (kcal/mol) and binding poses were generated for each ligand–protein pair. The best‐ranked poses were visualized using PyMOL (Schrödinger, LCC, 2021) and Discovery Studio Visualizer (Dassault Systemes BIOVIA, 2021).

To validate the docking protocol, the cocrystallized ligands of ERα (3ERT), AKT1 (3O96), AR (2Q7I), CDK2 (1PYE), and BCL‐2 (6O0K) were extracted and independently re‐docked into their respective binding sites. The re‐docked conformations closely reproduced the experimentally determined crystallographic poses, with root‐mean‐square deviation (RMSD) values below 2.0 Å, thereby confirming the reliability of the docking protocol [82, 83].

## Author Contributions

**Racha Lydia Bouchouka**: methodology, investigation, data curation, formal analysis, writing – original draft. **Farida Larit**: software, data curation, formal analysis, methodology, visualization, writing – original draft, writing – review and editing. **Zahia Kabouche**: conceptualization, data curation, investigation, methodology, project administration, supervision, visualization, Writing – original draft, writing – review & editing; **Laurent Riou**: methodology, writing – original draft, writing – review & editing. **Marlène Debiossat**: investigation, methodology, validation, writing – original draft, writing – review and editing. **Catherine Ghezzi**: investigation, methodology, writing – original draft, writing – review and editing. **Francisco León**: supervision, methodology, writing – review and editing. All authors have read and agreed to the published version of the manuscript.

## Funding

This research was funded by the DGRSDT‐MESRS (Algeria) and the Laboratoire Radiopharmaceutiques Biocliniques (LRB, France).

## Conflicts of Interest

The authors declare no conflicts of interest.

## Supporting information




**Supporting File**: cbdv71724‐sup‐0001‐SuppMat.docx

## Data Availability

The authors have nothing to report.
