# Supplementary material for: Phytochemical Analysis Using LC–MS/MS and Anticancer Activity of Asphodelus tenuifolius Roots: Cell Cycle Arrest, Apoptosis, and In Silico Insights
Source: Chem Biodivers. 2026 Sep 22;23(9):e71724. doi: 10.1002/cbdv.71724 (PMC13598352; doi:10.1002/cbdv.71724)
Supplement: Supplementary file 1 [file CBDV-23-e71724-s001.docx]

Chemistry & Biodiversity

**Supplementary Materials**

**Phytochemical Analysis Using LC–MS/MS and Anticancer Activity of *Asphodelus tenuifolius* Roots: Cell Cycle Arrest, Apoptosis, and In Silico Insights**

Racha Lydia Bouchouka^a,b^, Farida Larit^a^*, Zahia Kabouche^a^, Marlène Debiossat^b^, Laurent Riou^b^, Catherine Ghezzi^b^, Francisco Leónd^c^, Ahmed Kabouche^a,d^

^a^Université Constantine 1-Frères Mentouri, Laboratoire d’Obtention de Substances Thérapeutiques (LOST), Campus Chaabet Ersas, 25000 Constantine, Algeria

^b^Université de Grenoble Alpes, INSERM, CHU Grenoble Alpes, LRB, Grenoble, France.

^c^Department of Drug Discovery and Biomedical Sciences, College of Pharmacy, University of South Carolina, Columbia, South Carolina 29208, United States

^d^Université Constantine 1-Frères Mentouri, INATAA, RN79, 25000 Constantine, Algeria

Correspondence: Farida Larit (laritfarida@umc.edu.dz)

Abstract: *Asphodelus tenuifolius* Cav. (Asphodelaceae) is traditionally used in North African ethnomedicine for inflammatory and oxidative stress-related disorders. In this study, the hydroethanolic root extract was investigated by LC–HRMS/MS, leading to the identification of 58 metabolites, mainly flavonoids, anthraquinones, and cinnamic acid derivatives. Major constituents included luteolin, emodin, luteolin-7-*O*-β-D-glucoside, subaphyllin (*N*-*trans*-feruloylputrescine), and *N-trans*-feruloyltyramine. The extract showed moderate antiproliferative activity against MDA-MB-231 and PC-3 cells, while flow cytometry revealed pronounced apoptosis induction (up to 85%) and significant G0/G1 cell-cycle arrest. Molecular docking and target prediction suggested multitarget modulation of ERα, AR, AKT1, CDK2, and BCL-2, particularly by aglycone metabolites. ADMET analysis indicated favorable drug-likeness and safety profiles. These findings provide the first mechanistic evidence supporting the multitarget anticancer potential of *A. tenuifolius* roots and highlight their value as a source of bioactive anticancer scaffolds.

**Keywords**: *Asphodelus tenuifolius* • LC–HRMS/MS • Anticancer activity • Molecular docking • Apoptosis

**Contents**

**Figure S1.** Representative LC–ESI–DDA–HRMS base peak chromatogram of HERAT acquired in negative ionization mode.

**Table S1.** Two-way ANOVA Interaction Terms for Key Experimental Results

**Figure S1.** Representative LC–ESI–DDA–HRMS base peak chromatogram of HERAT acquired in negative ionization mode.

**Table S1.** Two-way ANOVA Interaction Terms for Key Experimental Results

| **Experiment** | **Factor A** | **Factor B** | **Interaction Term (A × B)** | **F-statistic** | **df (A × B)** | **p-value** | **Interpretation** |
| --- | --- | --- | --- | --- | --- | --- | --- |
| Cell viability (MTT assay) | Cell line | Treatment concentration | Yes | 5.23 | 1,16 | 0.034 | Significant interaction |
| Apoptosis (Annexin V/PI assay) | Dose | Time | Yes | 3.89 | 2,21 | 0.042 | Significant interaction |
| Cell cycle phase distribution | Dose | Cell cycle phase | No | 2.11 | 2,24 | 0.134 | Not significant |
